# A nanolaser with extreme dielectric confinement

**DOI:** 10.1126/sciadv.adx3865

**Published:** 2025-12-17

**Authors:** Meng Xiong, Yi Yu, Yury Berdnikov, Simon Klinck Borregaard, Adrian Holm Dubré, Elizaveta Semenova, Kresten Yvind, Jesper Mørk

**Affiliations:** ^1^ Department of Electrical and Photonics Engineering, Technical University of Denmark, Ørsteds Plads 345A, DK-2800 Lyngby, Denmark.; ^2^ NanoPhoton—Center for Nanophotonics, Ørsteds Plads 345A, DK-2800 Lyngby, Denmark.

## Abstract

The interaction between light and matter can be enhanced by spatially concentrating the light field and extending photon dwell time. Plasmonic structures can provide strong light confinement but suffer from ohmic losses. Recent advances in dielectric nanostructures enable strong light localization without metallic losses. However, previous studies primarily focused on minimizing the optical mode volume without adequately addressing light-matter interactions. Here, we demonstrate a nanolaser that colocalizes photons and excited carriers within a dielectric nanobridge. This extreme dielectric confinement of both light and matter yields a subdiffraction-limited mode volume and a subwavelength carrier volume without lateral quantum confinement. We observe a strong correlation between the mode field and carrier distribution, where enhanced mode localization produces stronger carrier confinement. By suppressing carrier surface recombination, this platform not only enables continuous-wave lasing at room temperature but also achieves a substantially reduced lasing threshold. We quantify the intensified interaction with an interaction volume, generalizing mode volume to a broad class of active media.

## Teaser

Nanolasers that strongly colocalize light and carriers in space enable ultraefficient continuous-wave lasing at room temperature.

## INTRODUCTION

The concentration and temporal storage of light in a cavity enhances light-matter interactions and underpins fundamental quantum optics and a myriad of applications, particularly lasers, which facilitate global connectivity by converting electrical signals into optical ones for long-distance communication. Present lasers, by electronic standards, are large and energy demanding, impeding their application for on-chip optical links to minimize losses from electrical interconnects (*1*–*5*). Such losses, exacerbated by the rise of artificial intelligence (*6*), escalate the global energy consumption (*7*). Therefore, developing ultraefficient microscopic lasers is imperative, which necessitates small cavities capable of strong spatial light compression while maintaining low losses.

Since Purcell’s (*8*) insight that an atom’s emission rate can be markedly enhanced in a small cavity concentrating the electromagnetic vacuum field, minimizing the optical mode volume (*V*_mod_) has been an important research direction. Metallic structures supporting plasmonic excitations (*9*–*12*) have realized mode volumes below the so-called diffraction limit (*10*), quantified by [λ/(2*n*)]^3^, where λ and *n* are the operating wavelength and refractive index. However, plasmonics suffer from ohmic losses resulting in low quality factors (*Q* factors) (*12*). Conversely, a new class of dielectric nanostructures featuring sharp-edged geometries can confine light into a subdiffraction-limit “hotspot” through electromagnetic boundary conditions while preserving high *Q* factors (*13*–*19*). Recent implementations in passive nanobeams and photonic-crystal (PhC) lasers have reached exceptionally small *V*_mod_ (*20*, *21*), but their mode fields are maximized in air, mirroring void structures (*22*–*28*). Thus, the light-matter interaction is compromised, necessitating, e.g., pulsed pumping to achieve lasing (*21*, *26*, *28*).

In this work, we demonstrate a nanocavity with extreme dielectric confinement (EDC) of both photons and electrons. We find that the mechanism used to spatially localize the optical field also concentrates the distribution of excited electrons. This colocalization, coupled with high gain and cavity *Q* factor, strongly enhances the interaction between light and matter. It enables the realization of a nanolaser with a mode volume below the diffraction limit and an ultralow threshold, operating at room temperature under continuous-wave (CW) pumping.

## RESULTS

### Interaction volume

While reducing *V*_mod_ is key for improving the efficiency of quantum light sources with a quantum emitter positioned at the antinode of the cavity mode field (*29*, *30*), this metric inadequately quantifies light-matter interactions in optoelectronic devices like lasers that typically contain many emitters distributed over a region larger than the mode volume. Merely reducing *V*_mod_ does not enhance light-matter interactions unless the “matter,” represented by carriers (electrons and holes), is also spatially localized. This necessity stems from the dependence of the light-matter interaction strength on the spatial overlap

$$\frac{1}{V_{I}}=\int N_{p}\left(r\right)N_{c}\left(r\right)dr$$ (1)

where *N*_p_(**r**) and *N*_c_(**r**) are the normalized photon and carrier densities. Here, we introduce the interaction volume (*V*_**I**_), a metric quantifying this overlap and reflecting the degree of spatial confinement for both photons and carriers. For a laser, the threshold power (*P*_th_) is proportional to the threshold carrier number (*n*_c,th_), which is proportional to *V*_**I**_ (section S4)

$$P_{\text{th}}\propto n_{c,\text{th}},\text{with}\;n_{c,\text{th}}=V_{I}\frac{\omega_{c}}{\mathrm{gQ}}+n_{\text{tr}}$$ (2)

Here, *n*_tr_ is the number of carriers needed to reach transparency, where stimulated absorption and emission exactly balance, and *V*_**I**_ω_c_/(*gQ*) is the additional number of excited carriers required to offset the cavity losses, thereby achieving lasing. The parameters ω_c_, *g*, and *Q* are the laser frequency, differential gain coefficient, and cavity *Q* factor, respectively. Equation 2 shows that the minimum laser threshold should target a minimized *V*_**I**_ rather than *V*_mod_, while simultaneously maximizing *Q* and *g*. In conventional lasers where the carrier density is relatively uniform across the active region, *N*_c_(**r**) can be factored out of the volume integral of Eq. 1. This reduces *V*_**I**_ to the optical volume used in traditional laser theories (*31*, *32*) (section S4.1), which is inversely proportional to the optical confinement factor (Γ). In the opposite limit of a dimensionless point dipole, *V*_**I**_ approaches *V*_mod_ (section S4.2). In this scenario, an ultrasmall active material (*33*–*35*) must be precisely positioned at the hotspot. Besides the difficulty of such nanoscale alignment, the imposed lateral quantum confinement restricts the total carrier number available (*n*_c,a_) for stimulated emission (*n*_c,a_ must be larger than *n*_c,th_), thereby limiting the available optical gain, which reduces the output power and possibly impedes lasing. Similarly, void structures, which can house quantum emitters inside the voids to provide gain, face the same challenges. In contrast, the EDC geometry with an extended active medium offers high gain without alignment issues. Furthermore, as we will show, *N*_c_(**r**) in the EDC cavity can vary considerably even without lateral quantum confinement, leading to a substantially reduced *V*_**I**_ while preserving a high gain and *Q*, thereby greatly reducing *P*_th_.

### Devices and lasing characteristics

**Fig. 1.**
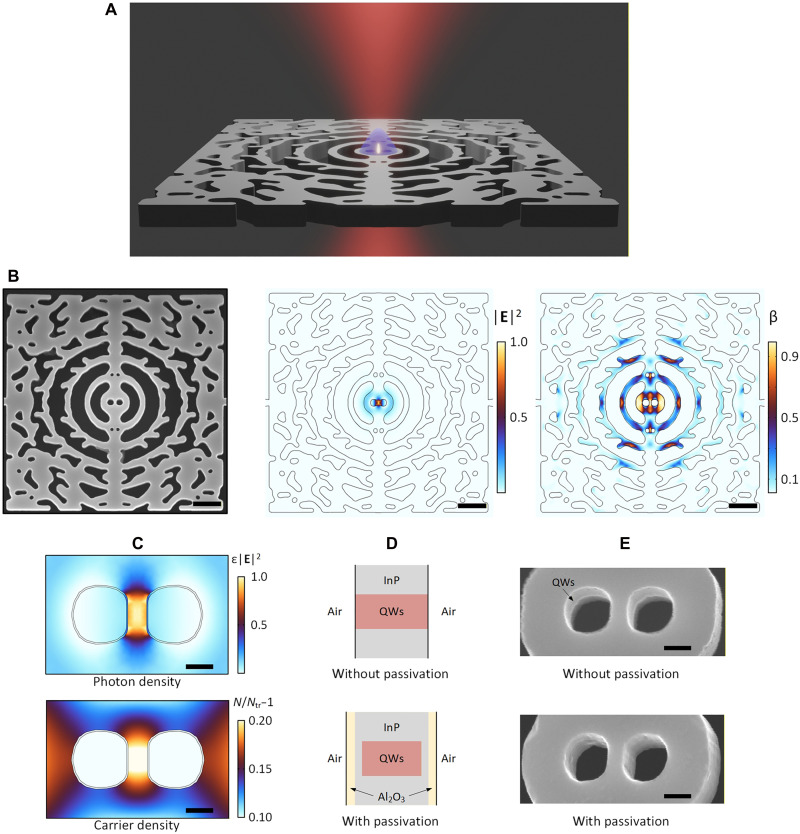
Nanolaser based on extreme dielectric confinement. (**A**) Schematic of an extreme dielectric confinement (EDC) laser under optical pumping with a focused Gaussian beam (red light), generating a region (blue shading) where excited carriers are concentrated and leading to enhanced spatial overlap with the optical cavity field (bright dot). (**B**) Left: Scanning electron microscope (SEM) image of a fabricated EDC laser based on an InP membrane embedded with quantum wells (QWs). Middle: Simulated lasing mode of the EDC cavity, showing that the electrical field (|**E**|^2^) is strongly localized at a hotspot within a central nanobridge, leading to an optical mode volume below the diffraction limit. Right: Corresponding calculated spontaneous emission factor (β), with a value approaching unity at the cavity center, attributed to the dominance of an EDC mode with small mode volume. The enhanced |**E**|^2^ and β suggest enhanced light-matter interactions when carriers are also concentrated at the cavity center. Scale bars, 1 μm. (**C**) Calculated photon (top) and carrier (bottom) density distribution at the cavity center, highlighting their colocalization at the same hotspot. *N* is the carrier density and *N*_tr_ is the transparency carrier density. This strong spatial carrier localization enhances surface recombination, necessitating surface passivation. The concentric “circles” depict a 5-nm Al_2_O_3_ layer used for surface passivation. Scale bars, 100 nm. (**D**) Schematics of the cross section of the central dielectric nanobridge without (top) and with (bottom) the surface passivation. The QWs (red) are first repaired and sealed with InP material (gray), then encapsulated by an Al_2_O_3_ layer (yellow) to mitigate the surface recombination. (**E**) SEM images of the central nanobridge without (top) and with (bottom) the surface passivation. Scale bars, 100 nm.

The EDC nanocavity (Fig. 1, A to C) features a mode with volume below the so-called diffraction limit, i.e., *V*_mod_ = 0.88[λ/(2*n*)]^3^, governed by a central dielectric nanobridge (80 nm width with 70 nm III-V materials). Topology optimization (*16*) is used for cavity design to maximize the *Q* factor while maintaining such a small *V*_mod_. However, topology optimization is not mandatory; the EDC structure can be adapted to various cavity configurations (*36*). Our specific cavity design is chosen to facilitate a fair comparison with a previous state-of-the-art nanolaser (section S3). With such a small *V*_mod_, the carrier distribution, rather than the mode profile, limits the light-matter interaction strength. Therefore, to keep a small *V*_**I**_ with a large optical confinement factor and avoid lateral quantum confinement (*37*), we do not minimize the bridge width (*19*) (section S5.3). To validate the strategy, we develop a laser model including photon and carrier spatial distributions (section S3). Simulations show central localization of both photons and carriers (Fig. 1E), ensuring large spatial overlap and a minimized interaction volume.

The structure is based on an InP membrane with embedded quantum wells (QWs). The fabrication follows the procedure in (*19*) with the notable addition of a surface passivation step involving wet etching for surface preparation, metal organic vapor phase epitaxy (MOVPE) annealing to reduce surface states and seal the surface, and Al_2_O_3_ deposition to further improve the sealing (Fig. 1D). These shield the QWs from air exposure and smoothens the sidewalls (Fig. 1E). Unlike conventional approaches that chemically treat the surface to remove dangling bonds and then encapsulate with dielectrics (*38*–*40*), our method directly seals QWs with InP using MOVPE annealing (Fig. 1D). This technique, similar to buried heterostructure technology (*33*–*35*), repairs crystal damage caused by ion bombardment during semiconductor dry etching. Additional chemical treatment of the InP sidewalls and final encapsulation with Al_2_O_3_ boost device stability and mitigate long-term degradation. This surface passivation is vital as it effectively suppresses surface recombination, a pervasive issue for nanostructures and particularly important for EDC lasers, where carriers are densely concentrated in space (section S2).

**Fig. 2.**
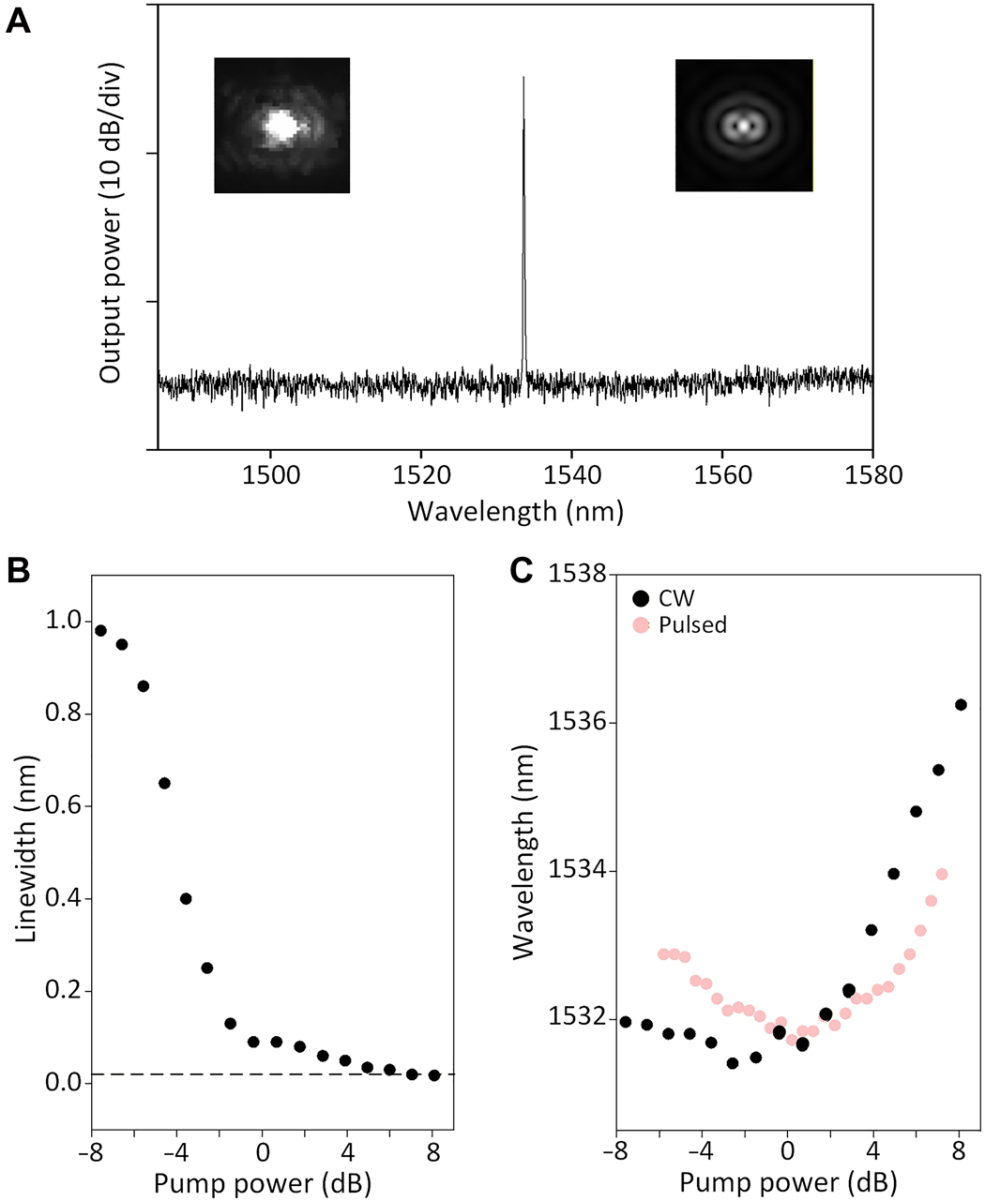
Continuous-wave, room-temperature lasing in the EDC laser. (**A**) Emission spectrum of the EDC laser above threshold, showing single-mode lasing. Insets: measured (left) and simulated (right) emission patterns. (**B**) Linewidth versus pump power, exhibiting a narrow linewidth limited at 0.02 nm (dashed line) by our spectrum analyzer at high pump powers. (**C**) Lasing wavelength versus pump power, showing a red shift above threshold. The shift is more pronounced under the CW (black) than pulsed (red) pumping. In (B) and (C), the pump power is normalized with 0 dB at the threshold point.

**Fig. 3.**
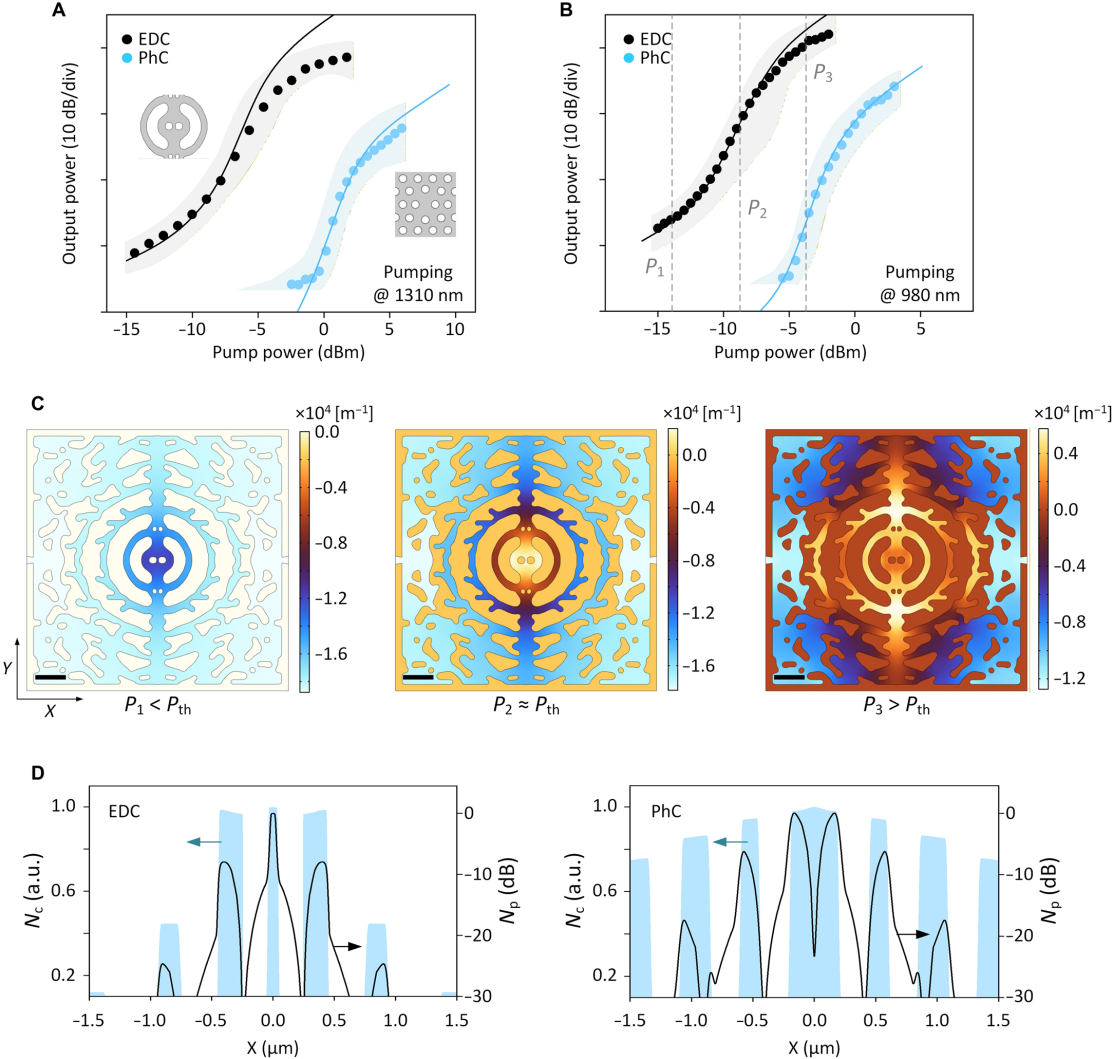
Threshold characteristics of the EDC laser. (**A** and **B**) Input-output characteristics for the EDC (black) and PhC (blue) laser under 1310 nm CW (A) and 980 nm pulsed (B) pumping. The inset in (A) shows schematics of the EDC (left) and PhC H0 (right) nanocavities. Experimental results (dots) align closely with theoretical predictions (solid lines), illustrating a lower threshold for the EDC than the PhC laser. The shaded area reflects the statistical variation among 20 nominally identical devices measured across the wafer. (**C**) Calculated EDC laser modal gain, which increases with carrier density: below (left), near (middle), and above (right) threshold, corresponding to pump powers of *P*_1_, *P*_2_, and *P*_3_, respectively, indicated by the dashed lines in (B). Scale bars, 1 μm. (**D**) Simulated photon (black curves) and carrier (blue shadings) density distributions at laser threshold under 980 nm pumping for the EDC (left) and PhC (right) laser along the *x* axis across the device center. The EDC laser exhibits much stronger field and carrier spatial localization than the PhC laser, with both maxima aligned at the cavity center. Values are normalized to their respective maxima.

The device is characterized by optical CW pumping at room temperature. The EDC laser exhibits single-mode lasing at 1535 nm (Fig. 2A), agreeing with simulations. Lasing is confirmed by the emission pattern (insets in Fig. 2A) and the narrow linewidth, limited at 0.02 nm by the optical spectrum analyzer (Fig. 2B). The input-output measurement displays a typical *S* curve but flattens at high pump powers (Fig. 3A), where the lasing wavelength red shifts (Fig. 2C) due to thermal effects. We also performed pulsed pumping at 980 nm, where the red shift is suppressed (red dots in Fig. 2C) (the lasers also work under 980-nm CW pumping).

### Spatial localization of electrons

To evaluate the performance of the EDC laser, we compare it to a reference nanolaser using an established point-defect cavity. Specifically, we choose the H0 point-defect cavity with the same footprint and fabricated using the same process, and a *V*_mod_ = 2.2(λ/2*n*)^3^, the smallest among PhC point-defect cavities (*41*, *42*). Additional rationale for selecting this PhC laser is provided in section S3.1. The total *Q* factors for the EDC and PhC lasers are estimated at ~6500 and 14,000, respectively. Despite expected higher perturbation sensitivity (*36*), the statistical variations of the EDC lasers are similar to the PhC lasers (shadings in Fig. 3, A and B), confirming good reproducibility using current nanofabrication technology. An important observation is that the EDC laser exhibits a threshold power density of only 5 kW cm^−2^, much lower than the PhC laser, despite its lower *Q* factor, smaller optical confinement factor (see section S3.2.2), and larger thermal red shift. We found that this behavior cannot be explained by conventional laser models; however, it is well reproduced in our simulations that include carrier localization effects (more details can be found in fig. S5). In these simulations, the modal gain (Fig. 3C), increasing with carrier density, is higher at the device center from below to near threshold, indicating that the lower threshold is correlated with a stronger central carrier concentration (Fig. 3D).

**Fig. 4.**
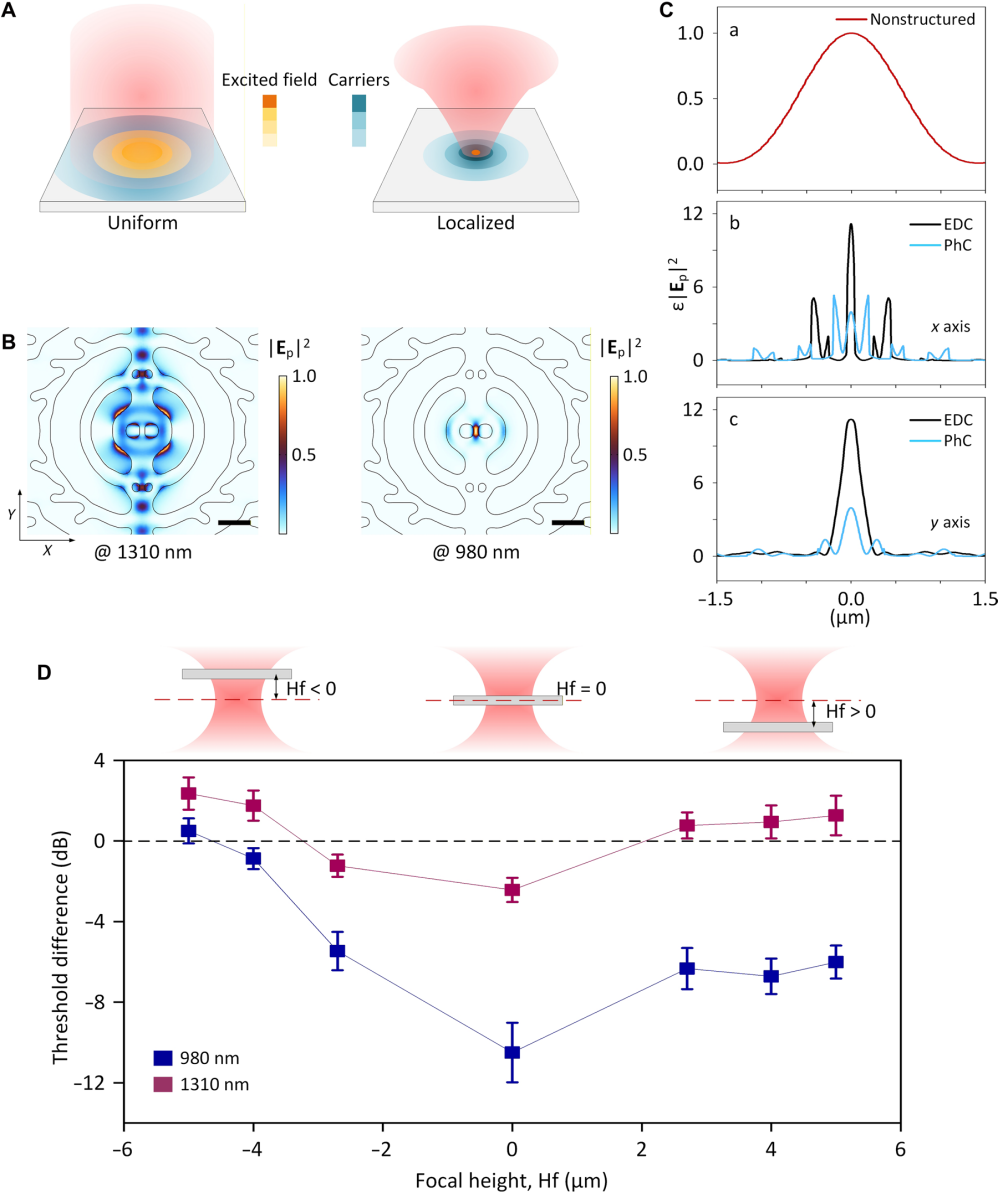
Reducing laser threshold via spatial carrier localization. (**A**) Pumping schemes: Uniform pumping (left) excites multiple cavity modes, resulting in a dispersed field pattern and evenly distributed carriers in space, while localized pumping (right) preferentially excites a mode with a small mode volume, inducing a concentrated carrier distribution. The orange and blue color bars represent the intensity of the excited field and carriers, respectively, with darker shades denoting higher intensities. (**B**) Simulated pump-excited field (|**E**_p_|^2^) at 1310 (left) and 980 (right) nm across the EDC laser’s central plane, pumped by a focused Gaussian beam as in our experimental setup. Values are normalized to the respective maximum. Scale bars, 0.5 μm. (**C**) Simulated pump-excited field energy density (ε|**E**_p_|^2^) in a nonstructured membrane (Ca), and in nanostructured cavities along the *x* axis (Cb) and *y* axis (Cc) across the center of the EDC (black) and PhC (blue) lasers. The 980-nm Gaussian beam is used for pumping. Values are normalized to the maximum in (Ca). ε|**E**_p_|^2^ is strongly enhanced at the cavity center in the EDC compared to both the PhC and nonstructured cases, attributable to the electromagnetic boundary conditions enforced by the sharp-edged features of the EDC structure. (**D**) Measured threshold difference between the EDC and PhC laser (Δ*P*_th_ = *P*_th,EDC_ − *P*_th,PhC_) at different pump focal positions. On the left (right), the pump focus is below (above) the membrane, corresponding to a negative (positive) focal height, Hf. Blue (purple) data represent Δ*P*_th_ for the 980 (1310)–nm pump, where |**E**_p_|^2^ is more (less) localized in the EDC than in the PhC laser. Error bars represent the SD of measurements across 20 nominally identical devices.

The spatial carrier distribution is shaped by the pumping profile (Fig. 4A). Contrary to what is observed in optical switches where a smaller *V*_mod_ leads to a faster diffusion of the carrier distribution (*43*, *44*), the smaller *V*_mod_ in the EDC laser enhances carrier localization at the optical hotspot, fostering a self-alignment of light and matter.

The primary mechanism is that although the pump is a Gaussian beam, the excited field (|**E**_p_|^2^) forms an intricate pattern (Fig. 4B) dictated by the electromagnetic boundary conditions and representing a superposition of multiple modes (see fig. S11). This leads to not only a more concentrated but also a stronger |**E**_p_|^2^ in the nanostructured environment than the nonstructured case (Fig. 4C). The 980-nm pattern, unlike the much less localized 1310-nm pattern, closely resembles the corresponding lasing mode, with much higher intensity at the EDC cavity center than the PhC (Fig. 4C). This self-alignment phenomena at shorter wavelengths stems from a smaller spot size and larger light wavevectors, facilitating the excitation of the mode with the smallest *V*_mod_ through improved spatial and momentum matching. In addition, our EDC cavity geometry, devoid of competing modes near the pump light frequency, further aids in exciting the lasing mode despite their considerable frequency separation.

Besides the direct benefits from the smaller *V*_mod_, the EDC structure offers additional advantages. Unlike bowtie nanostructures (*18*, *19*), the nanobridge restricts carrier diffusion by reducing the diffusion space from two-dimensional (2D) to quasi-1D (*43*, *44*). Moreover, the EDC cavity features a larger spontaneous emission factor (β) at the center than the outer regions (Fig. 1B) (note that, here, β refers to the optical β, not the overall β; see section S3.1). Therefore, unlike the usual case where stronger field localization increases the rate of stimulated emission and thereby burns a hole in the carrier distribution, the enhanced β, stemming from the smaller *V*_mod_, relatively reduces carrier loss to nonlasing modes, thus providing the possibility of intensifying the spatial carrier imbalance. Consequently, the heightened central carrier concentration boosts the spatially averaged β experienced by the overall carriers, further reducing the laser threshold.

To assess the carrier localization effect, we measure the laser thresholds by varying the pump focal plane. While the pump power in Fig. 3 represents preinjection levels, the power absorbed differs between structures due to different excitation patterns. For clarity, we normalize the input power to ensure equal energy absorption for both lasers (section S5.2). At the focal plane, the lasers reach their minimum thresholds, which increase as the focal plane shifts because their excitation patterns disperse (section S5.2). The EDC laser exhibits a lower threshold, with a reduction of up to 12 (3) dB at the focal plane under the 980 (1310)–nm pumping compared to the PhC laser (Fig. 4D). Notably, the EDC laser maintains a lower threshold at 1310 nm than the PhC laser despite its more diffused excitation pattern (fig. S8), highlighting its inherent superiority in carrier localization.

## DISCUSSION

Given the importance of carrier localization, we introduce the carrier volume $V_{\text{car}}=\int N_{c}\left(r\right)dr/\text{max}\left\{N_{c}\left(r\right)\right\}$ to quantify the spatial extent of matter. The EDC laser achieves a *V*_car_ of 0.28(λ/*n*)^3^ and 0.4(λ/*n*)^3^ at threshold under the focused 980- and 1310-nm pumping, compared to 2.1(λ/*n*)^3^ and 1.45(λ/*n*)^3^ for the PhC laser. This improvement far surpasses what is seen in *V*_mod_. The effective diameter of *V*_car_ (if treated as a disc) for the PhC laser is ~3.8 (3.2) μm under 980 (1310)–nm pumping, whereas it reduces to ~1.4 (1.7) μm in the EDC laser, which is even smaller than that of the pump beam, measured to be ~2 (2.5) μm.

To understand how the interaction volume *V*_**I**_ depends on *V*_mod_ and *V*_car_, we assume that *N*_p_(**r**) and *N*_c_(**r**) have in-plane Gaussian distributions. Then, *V*_**I**_ is found to be a combination of *V*_mod_ and *V*_car_ (section S4.3)

$$V_{I}=V_{\text{mod}}+V_{\text{car}}/\Gamma$$ (3)

Normally, *V*_mod_ is much smaller than *V*_car_ in nanocavities; reducing *V*_car_ is thus more important. Extending the analysis to more complex profiles beyond Gaussian would be an interesting direction for future work, although we do not expect it to alter the proportionality relationships between these volume metrics. From Eq. 1, the *V*_**I**_ for the EDC laser is 4.2(λ/*n*)^3^ and 5.5(λ/*n*)^3^ under 980- and 1310-nm pumping, compared to 31(λ/*n*)^3^ and 22(λ/*n*)^3^ for the PhC laser. These values align well with the estimates from Eq. 3. The results confirm that the EDC structure enhances carrier concentration in regions with the strongest mode field, effectively reducing *V*_**I**_, which, in turn, lowers the laser threshold.

We have demonstrated a room-temperature CW nanolaser exploiting extreme spatial localization of both light and excited electrons at the same hotspot. This self-alignment leads to enhanced light-matter interactions and a substantial reduction in the laser threshold. We introduce the light-matter interaction volume as the key metric to quantify this effect. Compared to other types of lasers, the EDC laser effectively manages the traditional trade-off between carrier volume and available electron states, similar to how it addresses the trade-off between mode volume and cavity *Q* factor. The EDC thus substantially lowers the interaction volume while maintaining high gain and cavity *Q* factor. This advancement is expected to elevate light-matter interactions to unprecedented high levels. The principle extends beyond light sources, affecting other optoelectronic devices (*45*–*49*), as well as sensing and imaging (*50*, *51*).

## METHODS

### Fabrication process

The device is fabricated within a 250-nm-thick InP membrane embedded with three InGaAsP QW layers. First, the InP wafer is flip bonded to a Si/SiO_2_ substrate. The laser membrane is formed after removing the InP substrate and an InGaAs etch-stop layer.

A SiN_*x*_ layer is deposited onto the wafer, followed by the spin coating of a photoresist. The structures are then patterned using electron beam lithography. This pattern is transferred to the SiN_*x*_ hard mask and subsequently to the InP layer through a two-step semiconductor etching. Following the etching, the sample undergoes a passivation process, where it is first treated in a solution of ammonium hydroxide to remove the oxide layer and then annealed inside the MOVPE reactor to repair and seal the QWs. After the annealing, the structures are membranized and encapsulated with an Al_2_O_3_ layer via atomic layer deposition. Further details are provided in section S2.

### Experimental setup

The samples are vertically pumped by a Gaussian light beam through an objective lens with a numerical aperture of 0.65 using a micro-photoluminescence setup. This setup uses three laser diodes (Thorlabs CLD1015) as pump sources, the full set of light sources available in our laboratory: a CW laser at 1310 nm, a CW laser at 980 nm, and a pulsed 980-nm laser. The laser is cascaded with an attenuator to adjust the pump power. Precise control over the pump position and area is maintained and monitored by an infrared camera (Xeva). The emission from the sample is collected vertically using the same objective lens. For the output measurements, after being isolated from the reflected pump beam by a long-pass filter, the collected signal is analyzed using an optical spectrum analyzer (YOKOGAWA AQ6370D) with a spectral resolution of 0.02 nm.

As for the pulsed pumping, we chose a pulse width of 0.2 μs with a duty cycle of 10%. This pulse width ensures that the transient regime, during which the laser reaches its steady state, is short compared to the pulse width so that the lasing state is maintained for an extended period. The low duty cycle effectively reduces thermal effects. To adjust the focal plane of the pump light, we tune the voltage of a piezo actuator (Thorlabs MDT693A) correlated with the *z* direction of an XYZ stage (NanoMax-TS) holding the sample. This stage provides a movement distance of 20 μm per 75 V applied.

### Numerical simulation and theory

The optical properties of the devices are calculated using both finite-difference time-domain (FDTD) simulations based on Ansys Lumerical FDTD and finite element method (FEM) calculations based on COMSOL Multiphysics. The two methods show good agreement. In addition, a 2D QW laser model incorporating spatial details of the optical mode field, pump-excited field pattern, spontaneous emission factor, and carrier transport is developed based on FEM using COMSOL Multiphysics. Further details on the model and underlying theory are provided in sections S3 and S4.

## Supplementary Materials

### This PDF file includes:

Sections S1 to S5 Tables S1 and S2 Figs. S1 to S16 References
