# Supplementary material for: A nanolaser with extreme dielectric confinement
Source: Sci Adv. 2025 Dec 17;11(51):eadx3865. doi: 10.1126/sciadv.adx3865 (PMC12710687; doi:10.1126/sciadv.adx3865)
Supplement: Supplementary file 1 — Sections S1 to S5 Tables S1 and S2 Figs. S1 to S16 References [file sciadv.adx3865_sm.pdf]

Science Advances
AAAS

Supplementary Materials for

A nanolaser with extreme dielectric confinement

Meng Xiong *et al.*

Corresponding author: Yi Yu, yiyu@dtu.dk; Jesper Mørk, jesm@dtu.dk

Sci. Adv. **11**, eadx3865 (2025)
DOI: 10.1126/sciadv.adx3865

This PDF file includes:

Sections S1 to S5
Tables S1 and S2
Figs. S1 to S16
References

S1. Nanolasers with sub-diffraction-limit mode volumes

Table S1 lists representative nanolasers with sub-diffraction-limit mode volumes. The extreme dielectric confinement (EDC) laser stands out with its high quality-factor (Q-factor), large gain and optical confinement factor, and more importantly, an ultrasmall interaction volume. It exhibits continuous-wave (CW) lasing at room-temperature.

Table S1. Nanolasers with sub-diffraction-limit mode volumes*

Laser type	Mode volume	Lasing wavelength	Threshold pump power density	Operating conditions
Plasmonic (52)	$0.000015\lambda^3$	373 nm	>50 $MW.cm^{-2}$	Pulsed (cryostat)
Plasmonic (53)	$0.0004(\lambda/(2n))^3$	870 nm	7 $GW.cm^{-2}$	Pulsed (RT)
Plasmonic (54)	$0.56(\lambda/(2n))^3$	1308 nm	120 $kW.cm^{-2}$	Pulsed (cryostat)
Metallic (55)	$0.5(\lambda/(2n))^3$	1354 nm	71 $kW.cm^{-2}$	Pulsed (RT)
Metallic (56)	$0.001\lambda^3$	1550 nm	20 $kW.cm^{-2}$	Pulsed (RT)
Dielectric void (21)	$0.0005\lambda^3$	1580 nm	26 $kW.cm^{-2}$	Pulsed (RT)
Dielectric void (26)	$0.003\lambda^3$	1550 nm	>2 $kW.cm^{-2}$	Pulsed (RT, in water)
EDC (this work)	$0.003\lambda^3$	1535 nm	5 $kW.cm^{-2}$	CW (RT)

*The table is compiled from literature reporting mode volumes below the diffraction limit ($(\lambda/(2n))^3$). Notably, a metallic nanolaser has demonstrated continuous-wave operation at room-temperature (57), but its mode volume of $5.88(\lambda/(2n))^3$ exceeds the diffraction limit. The pump power refers to the injected power before impinging on the device and is the peak-level power for pulsed pumping. For plasmonic/metallic lasers, the effective index (n) is often unspecified (e.g., Refs (53)–(55)) and depends on the mode profile. RT: room-temperature.

S2. Device and fabrication

The nanocavity design used for the EDC laser is taken from Ref. (16), and has been obtained through topology optimization. The spatial localization of the mode below the so-called diffraction-limited value of $(\lambda/(2n))^3$ can be explained as a consequence of the boundary conditions of the electromagnetic field at the interfaces between semiconductor and air (13), (14).

To avoid the extremely small features that typically arise in such optimization processes, a minimum length scale was imposed using a smoothed Heaviside projection filtering technique (16). The minimum feature size is primarily constrained by electron-beam lithography and semiconductor dry etching. For our InP platform, we found that this value should be larger than 50 nm; otherwise, etching through the structure becomes difficult. In this work, a filter radius of 96 nm was applied, enforcing a minimum length scale of 74 nm in both the dielectric and air regions using a geometrical constraint approach. Additionally, an 80 nm-wide non-designable InP region was introduced at the cavity center to ensure that the optical mode is confined within the solid rather than the air region. The bowtie is further surrounded by smooth, elliptic, grating-like regions. All features in the final design are smooth and conform to the imposed length-scale requirements.

Figure S1 illustrates the fabrication process. The device, with a ~7 μm×7 μm transverse footprint, is fabricated based on a 250 nm thick InP membrane, which in its middle comprises three InGaAsP/AlGaAs quantum wells (QWs). The InP wafer, with a 100 nm InGaAs etch-stop layer, is grown on an InP substrate via metal-organic-vapor-phase-epitaxy (MOVPE). Firstly, the wafer is flip-bonded to a Si/SiO_2 substrate. The Si/SiO_2 substrate is initially prepared through oxidation and annealing of a Si wafer in an anneal-oxide furnace at 1100 °C, yielding a 1.1 μm thick SiO_2 layer, which serves as a sacrificial layer for the membranization process. Before bonding, the surfaces of the InP wafer and the Si/SiO_2 substrate are activated with O_2 plasma for 30 seconds. The InP wafer is then directly bonded to the Si/SiO_2 substrate using a Süss SB6 wafer bonder at 300°C under a force of 2 kN. The InP substrate and InGaAs etch-stop layer are then removed to form the QW membrane.

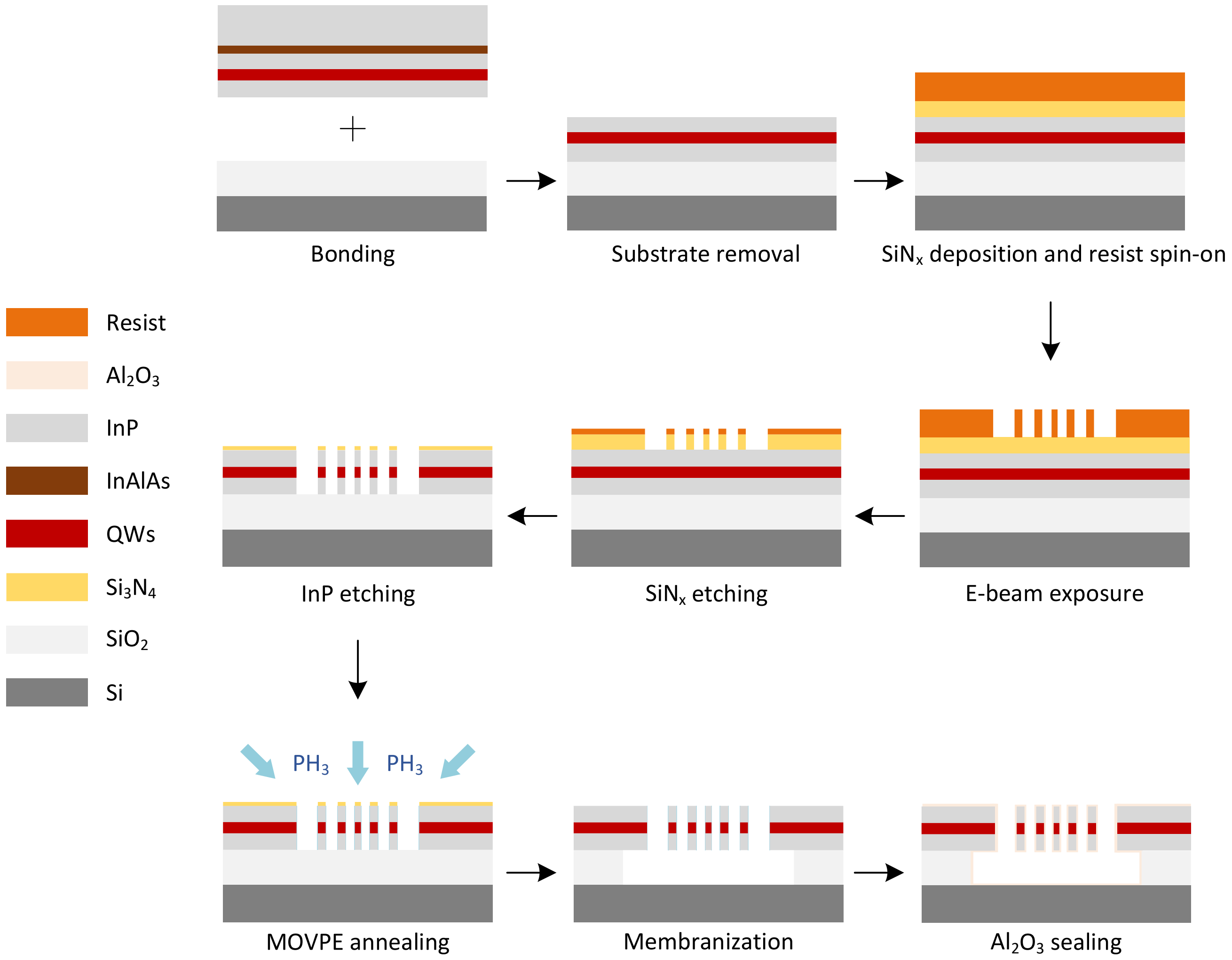

Fig. S1. Fabrication process. Schematic of our nanolaser fabrication process.

A 100 nm thick SiN_x layer is deposited onto the wafer, followed by a 180 nm thick chemically semi-amplified resist (CSAR 6200.09) via spin-coating. The SiN_x and CSAR layers serve as a hard mask and photoresist, respectively, with thicknesses optimized to allow etchant access to narrow cavity openings while protecting the underlying materials.

The laser structures are patterned using a JEOL-9500FSZ electron-beam writer with a 0.2 nA current and 1 nm shot pitch for high resolution. This pattern is first transferred to the SiN_x hard mask, then to the InP layer through a two-step inductively coupled plasma (ICP) etching. During ICP etching, the sample is mounted on a Si carrier wafer. This carrier wafer can form a passivation layer when interacting with the plasma, helping accumulate material on the sidewalls and effectively reducing lateral etching (19). For InP etching, HBr gas is employed at a

controlled flow rate of 5 sccm to maintain a low pressure of 0.5 mTorr. This low pressure is crucial for achieving straight sidewalls.

Following the etching, the sample undergoes passivation. The sample is first treated with ammonium hydroxide to remove the oxide layer formed on the sidewalls of the cavities during the etching and contact with the atmosphere. Then, the sample is annealed for 10 min under phosphine flux at 600°C inside the MOVPE reactor. At this temperature, annealing under phosphine initiates the replacement of arsenic atoms with phosphorus near the sidewall surface, thus isolating the QWs with a phosphorus-rich shell. After the annealing, the structures are membranized using a buffered hydrofluoric acid and then sealed with a 5 nm-thick layer of Al_2O_3 via thermal atomic layer deposition inside the Picosun R200 system. Compared to conventional methods that rely solely on chemical treatments followed by dielectric encapsulation (38)-(40), our surface passivation process incorporates an additional step of crystal quality repair and sealing of the InGaAsP QWs with "InP" through MOVPE annealing. This approach could be more effective at suppressing surface recombination (see section S3.2.3).

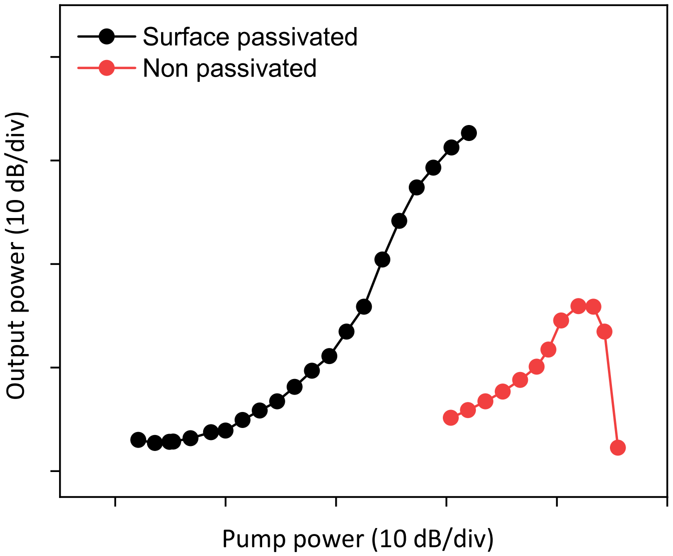

Fig. S2. Input–output characteristics of the EDC laser with and without surface passivation. Examples of measured input-output characteristics of EDC lasers with (black) and without (red) surface passivation. The abrupt decrease in output power at alleviated pump power levels in the non-passivated EDC laser is due to the damage of the central dielectric nanobridge.

Surface recombination is a common issue for nanostructures with high surface-to-volume ratios, leading to enhanced carrier losses and exacerbating heating problems. Prior experiments on similar EDC lasers without surface passivation required pulsed operation at room-temperature or CW operation at cryogenic temperatures. In contrast, the photonic-crystal (PhC) point-defect (H0-type) nanocavity lasers (and other types of PhC lasers) can exhibit CW lasing at room-temperature without surface passivation, though their performance improves with it. Room-temperature CW pumping of unpassivated EDC cavities either failed to produce lasing or caused permanent damage (Fig. S2), with subsequent inspections revealing burning of the dielectric nanobridge, in agreement with the strong carrier localization there.

S3. Numerical model

S3.1. Estimation of the spontaneous emission factor

For a quantum emitter at position $\mathbf{r}_0$ interacting with a cavity mode i, the emission decay rate is determined using Fermi's golden rule (58)

$$\Gamma_{e,i}(\mathbf{r}_0) = \frac{2\pi}{\hbar^2}\left|\mathbf{E}_i(\mathbf{r}_0)\cdot\vec{d}\sqrt{n_p+1}\right|^2 \int_{-\infty}^{\infty} d\omega D_c(\omega) L(\omega), \tag{S.1}$$

where $\vec{d}$ is the dipole moment of the quantum emitter, n_p is the total number of photons, $\mathbf{E}_i(\mathbf{r}_0) = E_i(\mathbf{r}_0)\vec{e}$ ($\vec{e}$ is the unit vector) is the vacuum electrical field, with an amplitude $E_i(\mathbf{r}_0) = \sqrt{\hbar\omega_c / \left(2\varepsilon(\mathbf{r}_0)V(\mathbf{r}_0)\right)}$, and $V(\mathbf{r}_0) = \int \varepsilon(\mathbf{r})\left|E_i(\mathbf{r})\right|^2 d\mathbf{r} \,/\left(\varepsilon(\mathbf{r}_0)\left|E_i(\mathbf{r}_0)\right|^2\right)$.

Here, $\varepsilon(\mathbf{r}) = \varepsilon_0\varepsilon_r(\mathbf{r})$ with $\varepsilon_r(\mathbf{r})$ being the relative permittivity. For spontaneous emission where $n_p = 0$, we can derive from Eq. (S.1) the following expression:

$$\Gamma_{e,i}(\mathbf{r}_0) = \frac{2\omega_c}{2\hbar\varepsilon(\mathbf{r}_m)V(\mathbf{r}_m)}\left|\vec{e}\cdot\vec{d}\right|^2 \frac{(\gamma_c+\gamma_e)/2}{(\omega_c-\omega_e)^2+\left((\gamma_c+\gamma_e)/2\right)^2}\frac{\left|E_i(\mathbf{r}_0)\right|^2}{\left|E_i(\mathbf{r}_m)\right|^2} = 2g_c^2\cos(\phi)\cos(\varphi)\rho(\omega_c). \tag{S.2}$$

In this formula, ω_c and Q are the resonant frequency and Q-factor of the cavity mode i, $\gamma_c = \omega_c / Q$ is the inverse of the cavity photon lifetime, γ_e represents the bandwidth of the emitter and $\omega_c - \omega_e$ is the detuning of ω_c with respect to the centre frequency of the emitter ω_e. The quantum emitter models the bandwidth and peak of our QW photoluminescence spectrum. The parameter $g_c = d\sqrt{\hbar\omega_c / \left(2\varepsilon(\mathbf{r}_m)V_{\text{mod}}\right)} / \hbar$ is the conventional coupling strength between the cavity mode and the quantum emitter, in which $\mathbf{r}_m$ is the position where $\varepsilon(\mathbf{r})\left|E_i(\mathbf{r})\right|^2$ is maximized, and $V_{\text{mod}} = \int \varepsilon(\mathbf{r})\left|E_i(\mathbf{r})\right|^2 d\mathbf{r} / \left(\varepsilon(\mathbf{r}_m)\left|E_i(\mathbf{r}_m)\right|^2\right)$ is the optical mode volume. The factor $\cos(\phi) = \left|\vec{e} \cdot \vec{d}\right|^2 / d^2$ accounts for imperfect alignment of quantum emitter polarization relative to the polarization of the local electrical field, and $\cos(\varphi) = \left|E_i(\mathbf{r}_0)\right|^2 / \left|E_i(\mathbf{r}_m)\right|^2$ accounts for spatial offset between the emitter and the mode field intensity maximum, and $\rho_e(\omega_c) = (\gamma_c + \gamma_e) / \left(2\left((\omega_c - \omega_e)^2 + \left((\gamma_c + \gamma_e)/2\right)^2\right)\right)$ accounts for detuning between the cavity spectrum and the active material.

Eq. (S.2) describes the decay rate of a single emitter. To derive the macroscopic decay rate, one needs to consider the QW local electronic density of states and the occupation probabilities of electrons and holes, determined by excited carrier density and, hence, the pump strength (31). Here, we use Eq. (S.2) as a reasonable approximation for moderate carrier density. While the specific form of the decay rate may vary, the ratios between different cavity modes typically remain more constant. Thus, we approximate the spontaneous emission factor (for mode i in 2D space) by

$$\beta_i(x, y) = \Gamma_{e,i}(x, y) / \sum_{n=1}^{\infty} \Gamma_{e,n}(x, y). \tag{S.3}$$

In our implementations, β_i is obtained numerically from a finite series of quasi-normal modes calculated within a 150 nm wavelength range centred on the targeted lasing mode i. It is important to note that in this study, β_i refers to the "optical" type, which excludes nonradiative recombination. It should be noted that the overall β-factor, including the effect of nonradiative recombination and thus lower than the optical β_i, can be determined from the

jump in the laser input-output curve around threshold (32), and is approximately 15% for the EDC laser and 5% for the PhC laser. Therefore, the EDC laser exhibits an improved β-factor compared to the PhC laser, despite the expected increase in carrier nonradiative recombination due to enhanced surface effects.

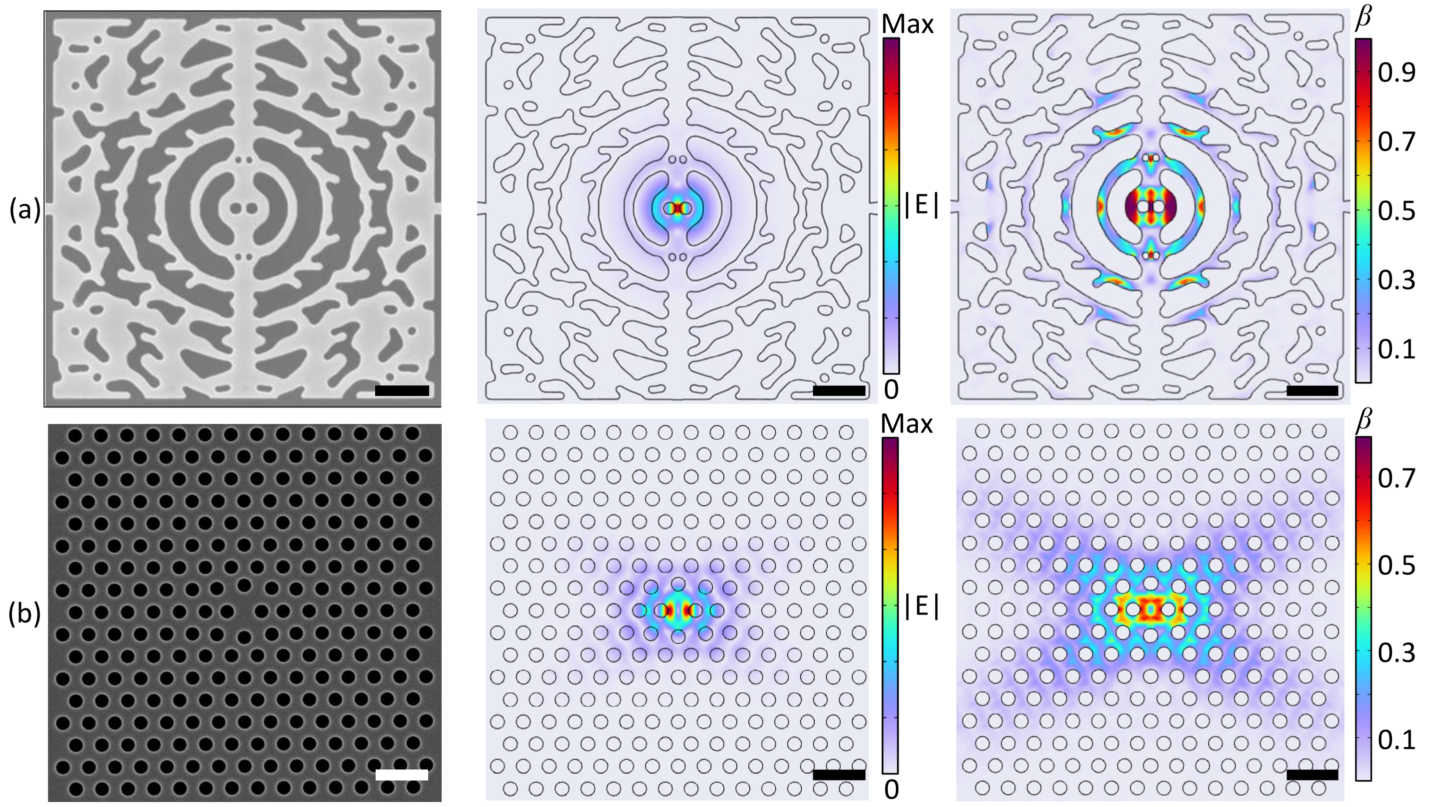

Fig. S3. Device structures, mode profiles, and spontaneous emission factors. (a, b) SEM images (left) of the EDC (a) and PhC H0 (b) cavities, along with the spatial profiles of their corresponding modes (middle) and spontaneous emission factors based on Eq. (S.3) (right). Scale bar: 1 μm.

The PhC H0 nanocavity is selected as a reference for its superior characteristics (59), including its small V_{mod} and high Q-factor for such a compact footprint, which are challenging to achieve with other designs. The simplest PhC H0 cavity is obtained by shifting the two neighboring holes at the center of the PhC structure. In this basic form, the cavity does not support a mode with high Q-factor. To enable a fair comparison with our EDC lasers, we optimized the pattern of holes near the center, following the standard approach (43). The PhC has the same membrane thickness as the EDC cavity (a thinner membrane typically leads to a smaller V_{mod}). Besides, both cavities lack modes near our pump light frequencies, ensuring that the pumping efficiencies are unaffected by

details of the mode pattern there. Additionally, the two devices have similar footprints, optical confinement factors, dielectric-to-air in-plane area ratios (impacting pumping efficiency), sidewall dielectric-air interface areas (influencing surface recombination), and comparable Q-factors.

Fig. S3 shows that the EDC cavity not only has a smaller V_{mod} but also a larger β_i at the centre compared to the PhC. This enhanced β_i is primarily due to only two major modes (modes having high intensity) at the EDC centre, whereas the H0 nanocavity features three modes. A smaller mode volume implies a larger free spectral range and fewer modes within a specified distance around the point where the mode volume is evaluated.

S3.2. Two-dimensional quantum-well laser model

We present our two-dimensional QW laser model, which, unlike previous efforts (60), incorporates spatial variations in the spontaneous emission factor, pump-induced excitation pattern along with carrier diffusion.

S3.2.1. Carrier rate equation

The carrier density $N_{ca}(\mathbf{r},t)$ in the QW can be separated as

$$N_{ca}(\mathbf{r},t) = N(x,y,t)N_z(z). \tag{S.4}$$

Here, $N(x,y,t)$ ($N_z(z)$) is the carrier density across the QW plane (along the QW growth direction). By assuming $N_z(z)$ uniform and normalized such that $N_z(z) = 1$, we get $N_{ca}(\mathbf{r},t) = N(x,y,t)$. The total carrier number, $n_c(t)$, is then given by

$$n_c(t) = \int N_{ca}(\mathbf{r},t)\,d\mathbf{r} = \int_z N_z(z)\,dz \iint_{xy} N(x,y,t)\,dxdy = h_{\mathrm{QW}} \iint_{xy} N(x,y,t)\,dxdy\,. \tag{S.5}$$

Here, h_{QW} is the total QW thickness. Our samples use three QWs, each about 7 nm thick. To avoid accounting for the vertical variations in field and carrier density, which is a minor effect, we consider a single effective active layer embedded at the device's central plane with an effective thickness of 20 nm.

The variable separation in Eq. (S.4) simplifies the problem to being two-dimensional, enabling us to focus solely on $N(x, y, t)$, which can be described by the following rate equation

$$\frac{d}{dt}N(x,y,t)=\frac{1}{q}\nabla\cdot J(x,y,t)-R(x,y,t)+G_p(x,y,t), \tag{S.6}$$

where

$$J(x,y,t)=q\mu N(x,y,t)\mathbf{E}_\text{b}(x,y,t)+qD\nabla N(x,y,t). \tag{S.7}$$

Here, μ and D are the carrier drift and diffusion coefficients, q is the electron charge, and $\mathbf{E}_\text{b}(x,y,t)$ is the background electrical field. Focusing on the laser steady-state, we adopt the ambipolar approximation (43), (44). Neglecting the drift effects, which are minor due to field screening by excited carriers, one gets $\nabla\cdot J(x,y,t)=qD\nabla^2 N(x,y,t)$.

In Eq. (S.6), $G_p(x,y,t)$ is the carrier generation rate, which scales with the pump power, and has a pattern determined by the pump source. $R(x,y,t)$ is the carrier recombination rate, which consists of stimulated emission, spontaneous emission, and nonradiative recombination, specifically

$$R(x,y,t)=R_{st}(N(x,y,t))+R_{sp}(N(x,y,t))+R_{nr}(N(x,y,t)). \tag{S.8}$$

The stimulated emission rate can be derived as

$$R_{st}(N(x,y,t))=G_N(N(x,y,t))n_p(t)\left|\mathbf{E}_{in}(x,y)\right|^2, \tag{S.9}$$

where we employ a QW gain model (31):

$$G_N(N(x,y,t))=\frac{\Gamma_z}{h_\text{QW}}\upsilon_g g_0\ln\left(\frac{N(x,y,t)+N_s}{N_{tr}+N_s}\right).$$

Here, $n_p(t)$ is the photon number, $\left|\mathbf{E}_{in}(x,y)\right|^2$ is the in-plane field energy density, Γ_z is the vertical (*z* direction) optical confinement factor. Their details are given in the next section. Additionally, υ_g is the material group velocity, g_0 is the material gain coefficient, N_{tr} is the transparency carrier density, and N_s is the so-called "linear parameter" employed to fit experimental data. The total spontaneous emission rate can be derived as

$$R_{sp}(N(x,y,t))=\frac{R_{sp,i}(N(x,y,t))}{\beta_i(x,y)}=\frac{1}{\beta_i(x,y)}R_N(N(x,y,t))\left|\mathbf{E}_{in}(x,y)\right|^2, \tag{S.10}$$

where an empirical formula (61) is used

$$R_N\left(N(x,y,t)\right)=\frac{1}{2}\frac{\Gamma_z}{h_{QW}}\upsilon_g g_0 \ln\left(1+\left(\frac{N(x,y,t)+N_s}{N_{tr}+N_s}\right)^2\right).$$

$R_{sp,i}\left(N\left(x,y,t\right)\right)$ is the spontaneous emission rate into the lasing mode (mode *i*), and $\beta_i\left(x,y\right)$ is the spontaneous emission factor presented in section S3.1. The nonradiative recombination can be expressed by

$$R_{nr}\left(N\left(x,y,t\right)\right)=\gamma_L N\left(x,y,t\right)+\gamma_A N\left(x,y,t\right)^3+R_{sr}\left(N\left(x,y,t\right)\right),$$

in which $\gamma_L N\left(x,y,t\right)$ represents the linear recombination and $\gamma_A N\left(x,y,t\right)^3$ reflects the Auger process. $R_{sr}\left(N\left(x,y,t\right)\right)$ denotes surface recombination, occurring exclusively at dielectric-air interfaces. This surface recombination necessitates the Neumann boundary condition: $D\nabla N\left(x,y,t\right)=-SN\left(x,y,t\right)$, where S is the surface recombination velocity.

S3.2.2. Photon rate equation

Similarly, the photon density N_{ph} may be decomposed as

$$N_{ph}(\mathbf{r},t)=n_p(t)\varepsilon(\mathbf{r})\left|\mathbf{E}(\mathbf{r})\right|^2\approx n_p(t)\varepsilon(x,y)\left|\mathbf{E}_z(z)\right|^2\left|\mathbf{E}_{xy}(x,y)\right|^2=n_p(t)\left|\mathbf{E}_z(z)\right|^2\left|\mathbf{E}_{in}(x,y)\right|^2.$$

We use $\varepsilon(\mathbf{r})\approx\varepsilon(x,y)$ given that the mode is typically well confined within the membrane and we neglect the difference between the refractive indices of the QW region and the cladding layers. A simplified notation, $\left|\mathbf{E}_{in}(x,y)\right|^2=\varepsilon(x,y)\left|\mathbf{E}_{xy}(x,y)\right|^2$ is adopted. The total photon number is

$$\int N_{ph}(\mathbf{r},t)d\mathbf{r}=n_p(t)\int N_p(\mathbf{r})d\mathbf{r}=n_p(t)\int_z\left|\mathbf{E}_z(z)\right|^2dz\iint_{xy}\left|\mathbf{E}_{in}(x,y)\right|^2dxdy=n_p(t). \tag{S.11}$$

The field has been normalized such that $\int_z \left|\mathbf{E}_z(z)\right|^2 dz = 1$ and $\iint_{xy} \left|\mathbf{E}_{in}(x,y)\right|^2 dxdy = 1$. The in-plane optical confinement factor is defined as $\Gamma_{xy} = \int_{xy\in\text{QW}} \left|\mathbf{E}_{in}(x,y)\right|^2 dxdy \,/ \int_{xy} \left|\mathbf{E}_{in}(x,y)\right|^2 dxdy$, which is found to be 0.93 (0.96) for our EDC (PhC H0) nanocavity. Noting that this definition of the optical confinement factor (where the integration region extends over the entire membrane containing QWs) is general and encompasses cases where the active region has defined lateral boundaries, as seen in conventional lasers with buried heterostructures (31). By employing the relations (z_w denotes the active layer in *z*, i.e., the centre plane of the membrane)

$$\int_{z\in\text{QW}} \left|\mathbf{E}_z(z)\right|^2 dz = \int_{z\in\text{QW}} \left|\mathbf{E}_z(z)\right|^2 dz \,/ \int_z \left|\mathbf{E}_z(z)\right|^2 dz \approx h_{\text{QW}} \left|\mathbf{E}_z(z_w)\right|^2 = \Gamma_z,$$

one gets $\left|\mathbf{E}_z(z_w)\right|^2 = \Gamma_z / h_{\text{QW}}$, and the dynamics of photon number can be derived as

$$\begin{aligned}\frac{d}{dt} n_p(t) &= -\gamma_c n_p(t) + \frac{h_{\text{QW}}}{\Gamma_z} n_p(t) \int G_N\left(N(\mathbf{r},t)\right)\varepsilon(\mathbf{r})\left|\mathbf{E}(\mathbf{r})\right|^2 d\mathbf{r} + \frac{h_{\text{QW}}}{\Gamma_z} \int R_N\left(N(\mathbf{r},t)\right)\varepsilon(\mathbf{r})\left|\mathbf{E}(\mathbf{r})\right|^2 d\mathbf{r} \\ &\approx -\gamma_c n_p(t) + h_{\text{QW}} n_p(t) \iint_{xy\in\text{QW}} G_N\left(N(x,y,t)\right)\left|\mathbf{E}_{in}(x,y)\right|^2 dxdy + h_{\text{QW}} \iint_{xy\in\text{QW}} R_N\left(N(x,y,t)\right)\left|\mathbf{E}_{in}(x,y)\right|^2 dxdy\end{aligned}.$$

(S.12)

Since $\varepsilon(\mathbf{r})\left|\mathbf{E}(\mathbf{r})\right|^2$ equals to N_p, and G_N and R_N are proportional to N (and $N_c=N/n_c$ as shown in the following section), the spatial integral in Eq. (S.12) is proportional to the volume integral of $\int N_p(\mathbf{r}) N_c(\mathbf{r}) d\mathbf{r}$, which is the inverse of the interaction volume to be discussed in Section S4.

S3.2.3. Implementation and parameters

Simulations are conducted as follows: First, the pumping profile $G_p(x,y)$ is calculated through FDTD (see section S5.1). Subsequently, the lasing mode and Q-factor were computed using FDTD and FEM (eigenmode solver). These simulated intrinsic Q-factors are consistent with the measured values obtained from scattering experiments on a similar passive sample. Then the β_i -values are calculated using FEM. These values are treated as constant in Eqs. (S.6) and (S.12), which are solved numerically using FEM. The parameters, comparable to those of conventional semiconductor QW lasers (31), are listed in Table S2. Notably, a surface recombination velocity of S = 2500 cm/s is extracted from theoretical fitting, much lower than the state-of-the-art values (38), (39). Ref. (40) achieved an ultra-low surface recombination velocity of only 260 cm/s, but this was with a much thicker dielectric capping layer of over 50 nm. The same study showed that this velocity rises to approximately 7000 cm/s with a thinner 5 nm (as in our case) dielectric capping layer.

Table S2. Parameter values used in simulations

Parameter	Symbol	Value
Refractive index	n	3.2(InP), 1.75(Al_2O_3)
Diffusion coefficient	D	5.75×10^{-4} $m^2.s^{-1}$
Surface recombination velocity	S	25 $m.s^{-1}$
Group velocity	υ_g	9.4×10^{7} $m.s^{-1}$
Total QW thickness (3 QWs)	h_{QW}	20 nm
Gain coefficient	g_0	1800 cm^{-1}
Transparency carrier density	N_{tr}	1.2×10^{24} m^{-3}
Carrier linear parameter	N_s	0.6×10^{24} m^{-3}
Linear recombination coefficient	γ_L	3.3×10^{7} s^{-1}
Auger recombination coefficient	γ_A	5×10^{-41} $m^6.s^{-1}$
Intrinsic Q-factor	Q_i	11000(EDC), 93000(PhC)
Absorption Q-factor	Q_a	16000
Total Q-factor	Q	$1/(1/Q_i+1/Q_a)$
Vertical optical confinement factor (3 QWs)	Γ_z	0.096

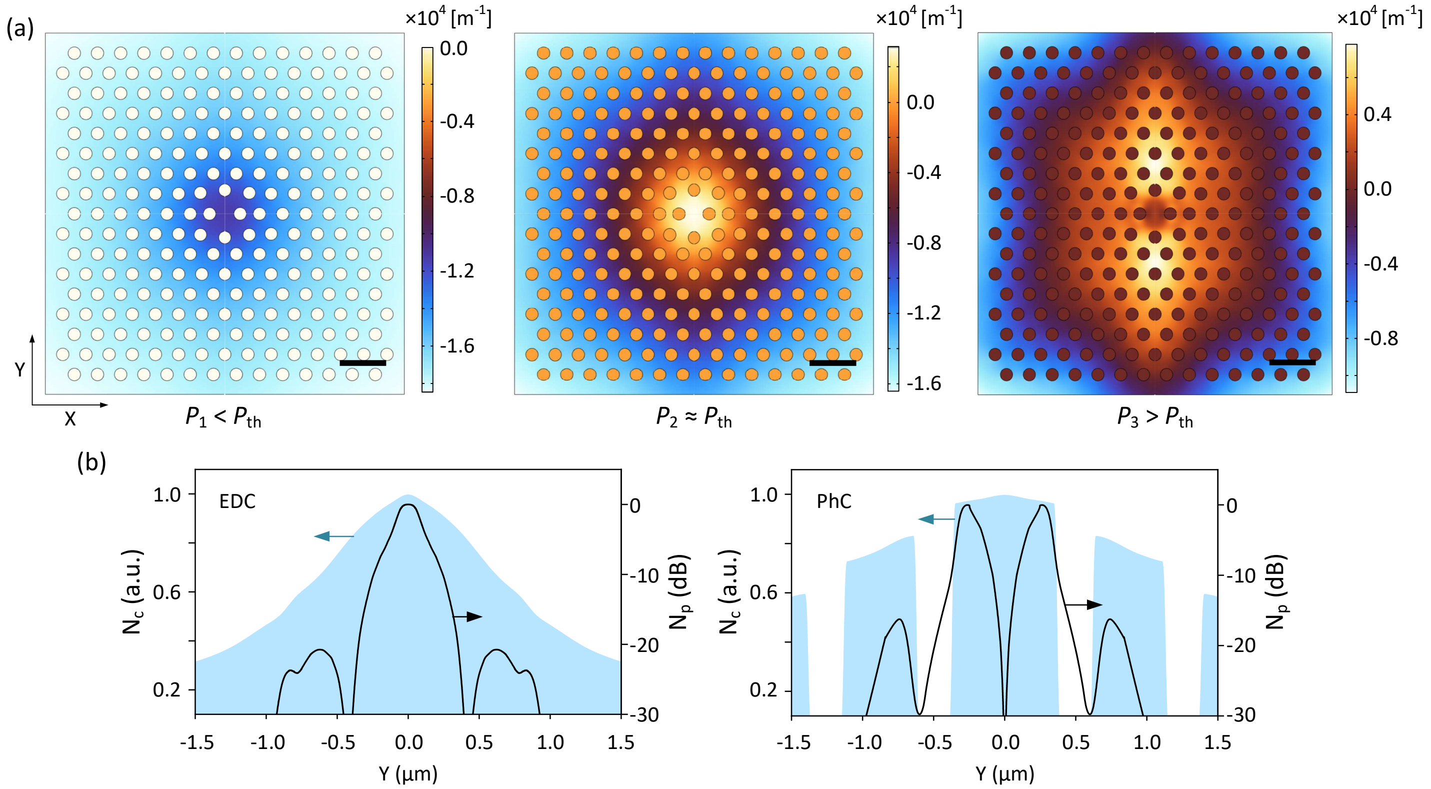

Fig. S4. Example of modal gain and carrier density distribution. (a) Calculated modal gain of the PhC H0 nanocavity laser at different power levels under 980 nm pumping: below (left), near (middle), and above (right) threshold. Scale bar: 1 μm. (b) Simulated photon (black curves) and carrier (blue shadings) density distributions at threshold under 980 nm pumping for the EDC (left) and PhC (right) lasers along the *y*-axis across the device centre. Values are normalized to their respective maxima.

Fig. S4 illustrates examples of the calculated modal gain and carrier density in the PhC laser. As seen, the modal gain is higher at the centre below and at threshold, but lower above threshold, which is attributed to spatial hole burning (62). Simulations show that a higher spontaneous emission factor at the centre relative to the outer region (see Fig. S3) boosts the central concentration of carriers.

The underlying mechanism involves the smaller mode volume of the EDC cavity, which reduces the likelihood of encountering competing mode with high field intensity within the "hotspot". This configuration funnels carriers directly into the lasing mode instead of losing them to non-lasing modes, thereby enhancing laser efficiency. Consequently, while carrier consumption is intensified at the center due to spatial hole burning above laser threshold, fewer carriers are consumed below to at threshold where the field intensity is weak. We find this promotes a higher central carrier concentration even without localized central pumping.

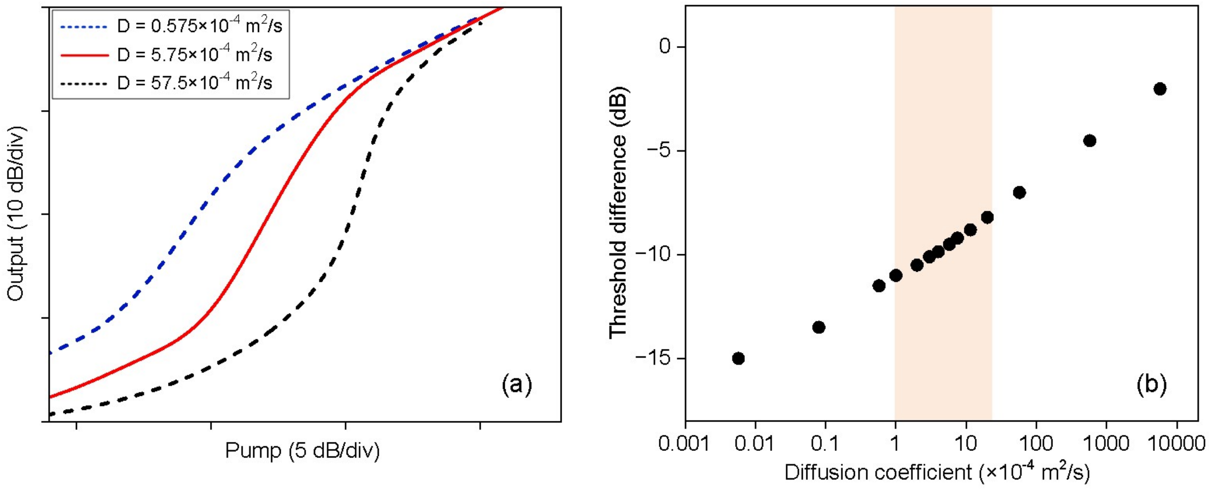

Fig. S5. Influence of carrier diffusion coefficient on the EDC laser threshold. (a) Simulated input-output curves of the EDC laser for different diffusion coefficients. (b) Threshold difference between the EDC and PhC lasers ($\Delta P_{th} = P_{th,EDC} - P_{th,PhC}$) as a function of diffusion coefficient, with the lasers being pumped at 980 nm at the focal plane. The shaded red region marks the typical range of diffusion coefficients for semiconductor InGaAsP quantum wells.

To test the validity of our model, we performed simulations with varied values for the diffusion coefficient D (Fig. S5). The results confirm that our conclusions hold within the standard range of values for D: a larger D increases the threshold and steepens the S-curve by reducing carrier localization, thereby lowering the spatially averaged β-factor. Nevertheless, the EDC laser consistently exhibits about an order of magnitude lower threshold within the typical range of D, consistent with the measurements. For very large diffusion coefficients, where carriers are effectively smeared out in space, this advantage tends to disappear. We notice that for the case of very large diffusion, the simulation requires a much enlarged domain to achieve convergence, which introduces technical challenges (e.g., within a limited simulation domain, the threshold curve becomes severely distorted, making it difficult to accurately predict the threshold).

We also performed simulations to investigate the robustness of our conclusions against variations of other important parameters, such as the Q-factor, surface recombination velocity, and differential gain. Again, these variations produced only minor changes in the results and did not affect our overall conclusion.

S4. Interaction volume, laser threshold, and carrier volume

We define the interaction volume $V_\mathbf{I}$ as a key metric for quantifying light-matter interactions:

$$V_\mathbf{I} = \frac{\left(\int N_c(\mathbf{r})d\mathbf{r}\right)\left(\int N_p(\mathbf{r})d\mathbf{r}\right)}{\int N_c(\mathbf{r})N_p(\mathbf{r})d\mathbf{r}} = \frac{1}{\int N_c(\mathbf{r})N_p(\mathbf{r})d\mathbf{r}}. \tag{S.13}$$

By employing the relations $N_p(\mathbf{r}) = \varepsilon(\mathbf{r})\left|\mathbf{E}(\mathbf{r})\right|^2$ and $\int N_p(\mathbf{r})d\mathbf{r} = 1$, and performing a Taylor expansion of the gain term, we get from Eq. (S.12) that at threshold

$$\gamma_c = \upsilon_g g_N \int \left(N(\mathbf{r}) - \tilde{N}_{tr}(\mathbf{r})\right) N_p(\mathbf{r}) d\mathbf{r}, \tag{S.14}$$

where $g_N = g_0 / \left(N_{tr} + N_s\right)$ is the laser differential gain, and

$$\tilde{N}_{tr}(\mathbf{r}) = N_{tr} + \frac{1}{2}\frac{\left(N(\mathbf{r}) - N_{tr}\right)^2}{N_{tr} + N_s} - \frac{1}{3}\frac{\left(N(\mathbf{r}) - N_{tr}\right)^3}{\left(N_{tr} + N_s\right)^2} + \dots. \tag{S.15}$$

Then, by applying the relationship $\int N(\mathbf{r})d\mathbf{r} = \int n_c N_c(\mathbf{r})d\mathbf{r} = n_c$, with $\int N_c(\mathbf{r})d\mathbf{r} = 1$ and n_c being the carrier number, and assuming $\tilde{N}_{tr}(\mathbf{r}) \approx n_{tr} N_c(\mathbf{r})$ so that $n_{tr} / V_\mathbf{I} = \int \tilde{N}_{tr}(\mathbf{r}) N_p(\mathbf{r}) d\mathbf{r}$, where n_{tr} is the total carrier number that must be inverted to reach transparency. The threshold carrier number is thus obtained as

$$n_{c,th} = V_\mathbf{I} \frac{\omega_c}{gQ} + n_{tr}, \tag{S.16}$$

which is proportional to $V_\mathbf{I}$. In Eq. (S.16), $\upsilon_g g_N$ is replaced by g for a more compact form. If taking the leading order in Eq. (S.15) (linearizing the gain term at threshold point), $n_{c,th}$ becomes $n_{c,th} = V_\mathbf{I}\left(\frac{\omega_c}{gQ} + \Gamma N_{tr}\right)$. This form may suggest that lowering $n_{c,th}$ requires a smaller optical confinement factor Γ. However, as we will show, $V_\mathbf{I}$ is inversely proportional to Γ, so a larger Γ is still preferable.

It needs to be noted that the carrier number required to reach threshold must be smaller than the available states within the homogeneous bandwidth (63) and thus depends on the electron density of states of the active material $\rho(E)$, i.e., one should fulfil the relation

$$n_{c,a} = \min\left\{V_a, V_{pump}\right\} \times \left(\int \rho(E) f(E) dE\right) > n_{c,th}, \tag{S.17}$$

so that the needed gain can be provided. Here, $n_{c,a}$ is the maximum carrier number available, $f(E)$ is the Fermi-Dirac distribution, capped by the maximum pump strength realistically achievable, $\min\{V_a, V_{pump}\}$ represents the smaller value of the active region volume V_a and the pumping volume V_{pump}. Satisfying Eq. (S.17) becomes challenging when V_a is small and $\rho(E)$ transits from a continuous to a more discretized distribution with respect to energy (E). This situation arises when lateral quantum confinement is introduced, e.g., with an ultrasmall buried heterostructure (35) or quantum dots are used as the active material.

We now relate $V_\mathbf{I}$ with the laser threshold power. As for the carrier rate equation, integrating both sides of Eq. (S.6) leads to the following equation at threshold

$$0 \approx \int D\nabla^2 N(\mathbf{r}) d\mathbf{r} - \int R_{sp}\left(N(\mathbf{r})\right) d\mathbf{r} - \int R_{nr}\left(N(\mathbf{r})\right) d\mathbf{r} + \int G_p(\mathbf{r}) d\mathbf{r}. \tag{S.18}$$

Here, we assume the net carrier flow outside the device is negligible, i.e., $\int D\nabla^2 N(\mathbf{r}) d\mathbf{r} \to 0$. On the other hand, one can replace it with an effective carrier loss, which can be absorbed into the nonradiative recombination term. By doing so, we arrive at

$$\int R_{sp}\left(N(\mathbf{r})\right) d\mathbf{r} + \int R_{nr}\left(N(\mathbf{r})\right) d\mathbf{r} \approx \int G_p(\mathbf{r}) d\mathbf{r} = \gamma_{th}, \tag{S.19}$$

where $\gamma_{th} = \int G_p(\mathbf{r})d\mathbf{r} = P_{th} / (\hbar\omega_c)$ is the laser threshold pump rate. Since $R_{sp}(N(\mathbf{r})), R_{nr}(N(\mathbf{r})) \propto N(\mathbf{r})$, with $N(\mathbf{r}) \propto n_{c,th}$, the laser threshold power P_{th} is proportional to $n_{c,th}$, and consequently, to $V_{\mathbf{I}}$, i.e.,

$$P_{th} \propto V_{\mathbf{I}} \frac{\omega_c}{gQ} + n_{tr} . \tag{S.20}$$

As seen, a smaller $V_{\mathbf{I}}$ reduces the number of carriers needed to offset laser losses, thereby lowering the threshold power. As we can also find from Eq. (S.10), R_{sp} is proportional to $1/\beta_i$. Since $\beta_i(\mathbf{r})$ is larger at the "hotspot" (see Fig. S3), a carrier distribution concentrated at the field maximum enhances the spatially-averaged spontaneous emission factor, further reducing P_{th}. In the following, we analyze the interaction volume $V_{\mathbf{I}}$ in different regimes.

S4.1. Case 1: Carrier distribution varies much slower than photon distribution

If the carrier density varies slowly in space where the photon distribution peaks, $N(\mathbf{r})$ can be considered constant in the volume integral of Eq. (S.13). Consequently, $N(\mathbf{r} =)n_c N_c(\mathbf{r} \approx)n_c N_c$. When considering a finite lateral dimension of the active region, as in a laser with a buried heterostructure, we obtain

$$\int N(\mathbf{r})d\mathbf{r} = n_c N_c \int_{\text{active}} d\mathbf{r} = n_c N_c V_a = n_c \Rightarrow N_c = \frac{1}{V_a}, \tag{S.21}$$

where V_a is the volume of the active region. In this case, we find

$$\frac{1}{V_{\mathbf{I}}} = \int N_c(\mathbf{r}) N_p(\mathbf{r}) d\mathbf{r} = N_c \int_{\text{active}} N_p(\mathbf{r}) d\mathbf{r} = N_c \frac{\int_{\text{active}} N_p(\mathbf{r}) d\mathbf{r}}{\int N_p(\mathbf{r}) d\mathbf{r}} = N_c \Gamma = \frac{1}{V_a / \Gamma} = \frac{1}{V_{op}} . \tag{S.22}$$

Eq. (S.22) shows that $V_{\mathbf{I}}$ reduces to the conventional optical volume $V_{op} = V_a/\Gamma$ used in modelling traditional light sources (31). Therefore, if neglecting the spontaneous emission, the threshold pump rate (Eq. (S.19)) becomes

$$\gamma_{th} \approx \gamma_{nr} V_a \left(\gamma_c / (\Gamma \upsilon_g g_N) + N_{tr}\right), \tag{S.23}$$

and the threshold power, $P_{th} = \hbar\omega_c \gamma_{nr} V_a \left(\gamma_c / (\Gamma \upsilon_g g_N) + N_{tr}\right)$, recovers to the form of macroscopic lasers.

S4.2. Case 2: Photon distribution varies much slower than carrier distribution

In the opposite case, where a single quantum emitter or a very small region of active material interacts with a slowly varying cavity mode in space, $N_p(\mathbf{r})$ can be assumed uniform across the integral in Eq. (S.13) so that

$$\frac{1}{V_{\mathbf{I}}} = \int N_c(\mathbf{r}) N_p(\mathbf{r}) d\mathbf{r} = N_p(\mathbf{r}_0), \tag{S.24}$$

where $\mathbf{r}_0$ is the spatial position of the quantum emitter. At the same time, we have

$$V_{\text{mod}} = \frac{\int N_p(\mathbf{r}) d\mathbf{r}}{N_p(\mathbf{r}_0)} = \frac{1}{N_p(\mathbf{r}_0)} \Rightarrow \frac{1}{V_{\text{mod}}} = N_p(\mathbf{r}_0). \tag{S.25}$$

Comparing Eqs. (S.24) and (S.25) reveals that $V_{\mathbf{I}}$ becomes the mode volume V_{mod} commonly used for quantum light sources. For a quantum emitter with a single electron in the excited state ($n_c = 1$), the spontaneous emission rate becomes

$$\int R_{sp}(N(\mathbf{r})) d\mathbf{r} = \gamma_{sp,i}(\mathbf{r}_0) + \sum_{n=1; n\neq i}^{\infty} \gamma_{sp,n}(\mathbf{r}_0).$$

In this situation, we arrive at the pumping rate required for driving single-photon sources, which operate in the spontaneous emission regime:

$$\gamma_{th} = \sum_{n=1}^{\infty} \gamma_{sp,n}(\mathbf{r}_0) + \gamma_{nr}. \tag{S.26}$$

S4.3. General situation

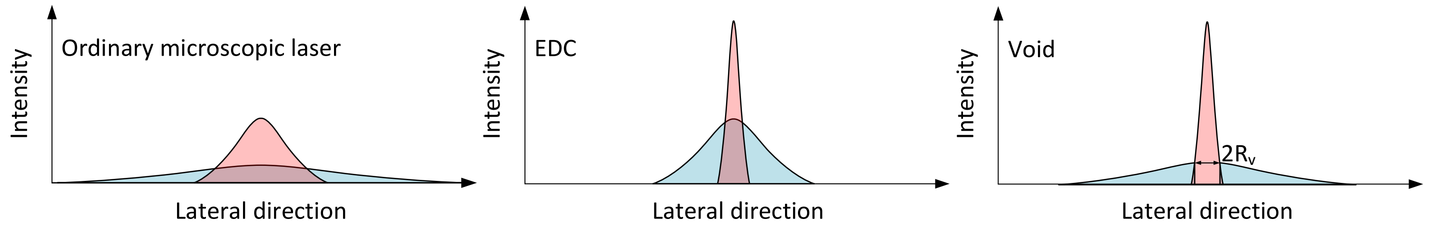

Fig. S6. Photon and carrier distributions for three representative scenarios. Schematics illustrating the spatial distributions of photons (red) and carriers (blue) with Gaussian distributions and overlapping maxima for a conventional microscopic laser without carrier confinement (left), EDC laser (middle), and a nanolaser with a void (air gap) region (right). Notice, that in the latter case, there are no carriers present at the spatial position where the cavity field is maximized, since this is in the region of an air gap.

In general, V_I depends on both V_mod and the carrier volume V_car, which we define as $V_\mathrm{car} = \int N(\mathbf{r})d\mathbf{r} / \max\{N(\mathbf{r})\}$. We consider a simple scenario where both the photon $N_p(\mathbf{r})$ and carrier $N_c(\mathbf{r})$ density have Gaussian distributions in the lateral (x-y) plane but are uniform along the z direction (Fig. S6). In polar coordinates, $N_p(\mathbf{r}) = N_{p0}\exp\left(-r^2/\left(2\sigma_p^2\right)\right)$, $N_c(\mathbf{r}) = N_{c0}\exp\left(-r^2/\left(2\sigma_c^2\right)\right)\Theta\left(r^2 - R_v^2\right)$, where $r^2 = x^2 + y^2$ and $\Theta\left(r^2 - R_v^2\right)$ represents a step function accounting for the absence of active material in void regions, as relevant for structures where photon distribution has its maximum in air gaps (20), (21), (26). This void region is assumed to have a disc area of a radius R_v at the centre (see Fig. S6), so R_v=0 corresponds to cases without the void region. $W_{p,c} = 2\sqrt{2\ln 2}\sigma_{p,c}$ is the full-width-at-half-maximum (FWHM) of the respective Gaussian profiles (both have their maxima at the centre).

These Gaussian functions have been normalized such that $\int N_{p,c}(\mathbf{r})d\mathbf{r} = 1$. In this case, one can deduce that $N_{p0} = 1/\left(2\pi h\sigma_p^2\right)$ and $N_{c0} = 1/\left(2\pi h_\mathrm{QW}\exp\left(-R_v^2/\left(2\sigma_c^2\right)\right)\sigma_c^2\right)$, where h is the membrane thickness. The mode and carrier volume are obtained as $V_\mathrm{mod} = 1/N_{p0} = 2\pi h\sigma_p^2$ and $V_\mathrm{car} = 1/\left(N_{c0}\exp\left(-R_v^2/\left(2\sigma_c^2\right)\right)\right) = 2\pi h_\mathrm{QW}\sigma_c^2$, respectively. V_I can thus be derived as

$$V_\mathrm{I} = 1/\left(\int N_c(\mathbf{r})N_p(\mathbf{r})d\mathbf{r}\right) = \left(V_\mathrm{mod} + V_\mathrm{car}/\Gamma_z\right)\exp\left(R_v^2/\left(2\sigma_p^2\right)\right). \qquad \text{(S.27)}$$

Here $\Gamma_z \approx h_{\mathrm{QW}} / h$ is the vertical optical confinement factor. Simultaneously, the in-plane confinement factor becomes

$$\Gamma_{xy} = \frac{\int_0^{2\pi}\int_0^{\infty} N_p(r,\theta) r dr d\theta - \int_0^{2\pi}\int_0^{R_v} N_p(r,\theta) r dr d\theta}{\int_0^{2\pi}\int_0^{\infty} N_p(r,\theta) r dr d\theta} = \exp\left(-\frac{R_v^2}{2\sigma_p^2}\right). \quad (S.28)$$

Using Eq. (S.28) in Eq. (S.27) leads to

$$V_{\mathbf{I}} = \frac{V_{\mathrm{mod}} + V_{\mathrm{car}} / \Gamma_z}{\Gamma_{xy}} = \frac{V_{\mathrm{mod}}}{\Gamma_{xy}} + \frac{V_{\mathrm{car}}}{\Gamma}, \quad (S.29)$$

where $\Gamma = \Gamma_{xy}\Gamma_z$ is the total optical confinement factor. Eq. (S.28) shows that Γ_{xy} in a void/slot structure where $R_v \geq 0.5 W_c$ is no larger than 0.5, consistent with the results in Ref. (28). In contrast, in non-void cases, one typically has $\Gamma_{xy} \to 1$. Then, the interaction volume is reduced to

$$V_{\mathbf{I}} = \left(V_{\mathrm{mod}} + V_{\mathrm{car}} / \Gamma_z\right) / \Gamma_{xy} \approx V_{\mathrm{mod}} + V_{\mathrm{car}} / \Gamma . \quad (S.30)$$

As revealed, V_{car}/Γ plays a role similarly to the optical volume V_{op} for macroscopic lasers (31). Eq. (S.30) illustrates that when $V_{\mathrm{mod}} \ll V_{\mathrm{car}}$ ($V_{\mathrm{mod}} \gg V_{\mathrm{car}}$), $V_{\mathbf{I}}$ aligns with the scenarios described in section S4.1 (D.2).

S5. Additional data

S5.1. Pump-excited field patterns

Fig. S7 shows the schematic of the experimental setup for nanolaser characterization. The nanolaser is optically pumped through a microscope objective, with pump power controlled by an attenuator. Three pump sources (all available light sources in our lab) are used: a 1310 nm CW laser, a 980 nm CW laser, and a 980 nm pulsed laser. Pump position and area are accurately adjusted and monitored with an infrared camera. Emission is collected vertically by the same objective and, after isolation from the reflected pump beam with a long-pass filter, analyzed using an optical spectrum analyzer (0.02 nm resolution). The pump focal plane is tuned in the z direction using a piezo actuator coupled to an XYZ translation stage.

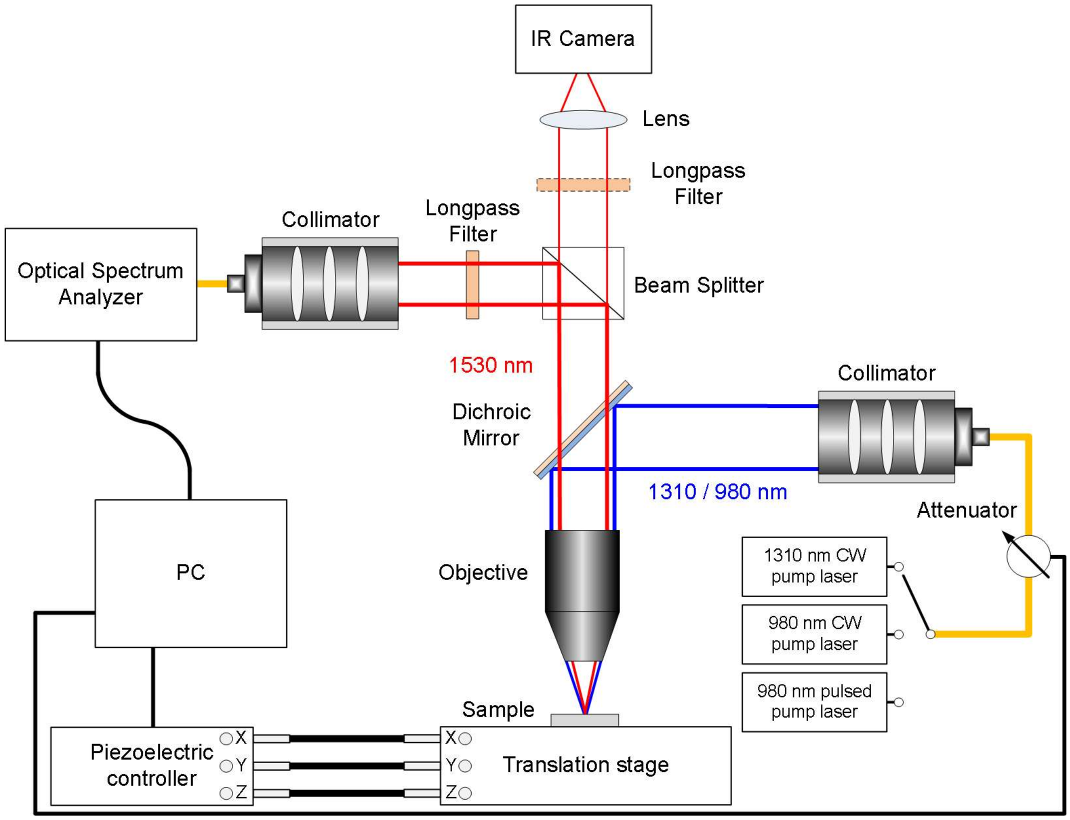

Fig. S7. Experimental setup. Schematic of our experimental setup for nanolaser characterization.

We analyze the field patterns excited by the pump beam using FDTD simulations. A pump laser beam at 1310 (980) nm with a beam diameter of ~2.5 (2.2) mm passes through an objective lens with a numerical aperture of 0.65, a lens diameter of 5.2 mm, and a focal length of approximately 4 mm. This setup produces a Gaussian beam with a $1/e^2$ spot diameter of ~2.5 (2) µm at the focal plane, which agrees very well with our measured values. The simulations encompass the vectorial nature of the Gaussian beam and the entire device structure, including the InP membrane and Si substrate, with the pump light polarized along the y-direction to maximize efficiency. Despite the initial Gaussian profile, the excited field ($|\mathbf{E}_p|$) across the QW plane exhibits a more complex pattern, cf. Figs. S8-S10. This pattern ($|\mathbf{E}_p|^2$, due to the dominant linear absorption) is subsequently imported as $G_p(x, y)$ into Eq. (S.6) for laser simulations.

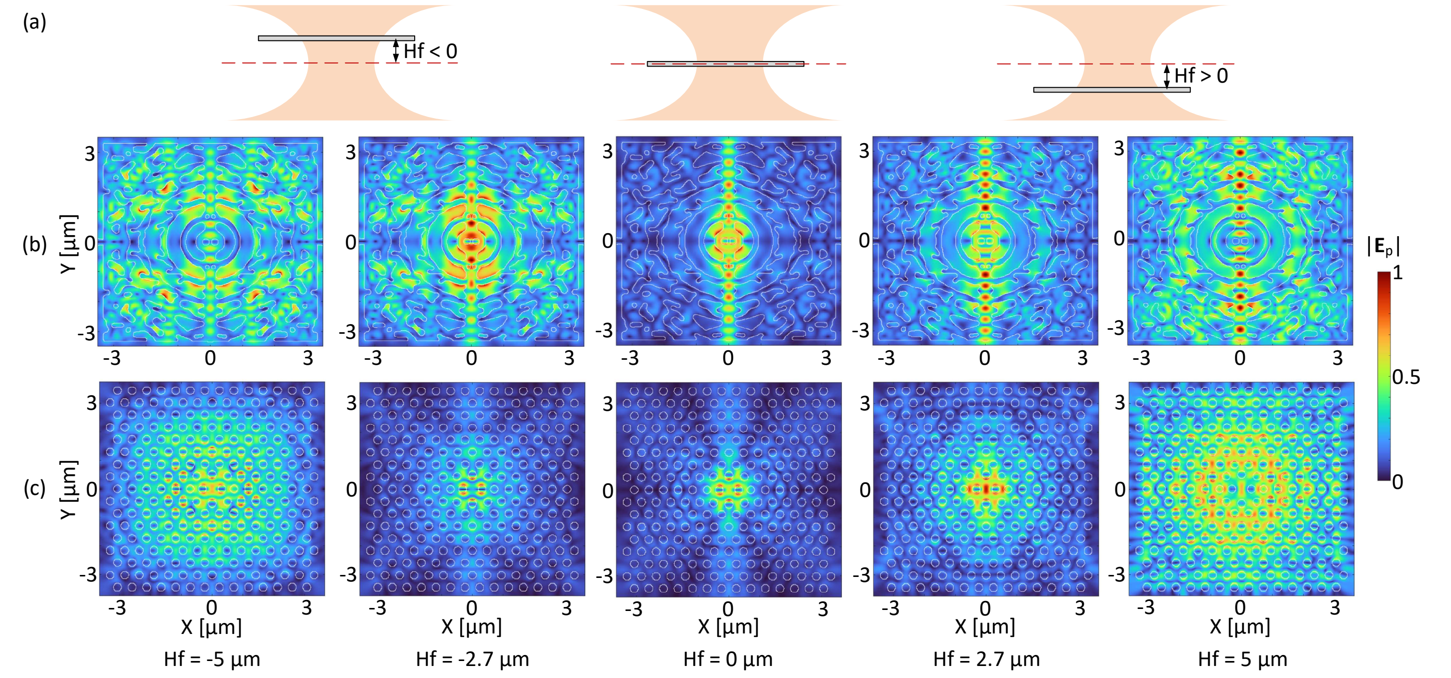

Fig. S8. Excitation profile of the pump beam at 1310 nm. (a) Schematic of the excitation, where a 1310 nm Gaussian beam (orange shading) from an objective lens strikes the membrane (gray slab) containing the EDC or PhC nanocavity. Dashed red lines indicate the focal plane of the Gaussian beam. The left (right) plot shows a negative (positive) focal height, Hf, with the focus below (above) the membrane, and the middle plot represents Hf=0. (b) Simulated pump excitation patterns (|$\mathbf{E}_\mathrm{p}$|, normalized) at the central plane of the EDC membrane. (c) Same as (b) but for the PhC nanocavity. The plots correspond to Hf ranging from -5 μm to 5 μm.

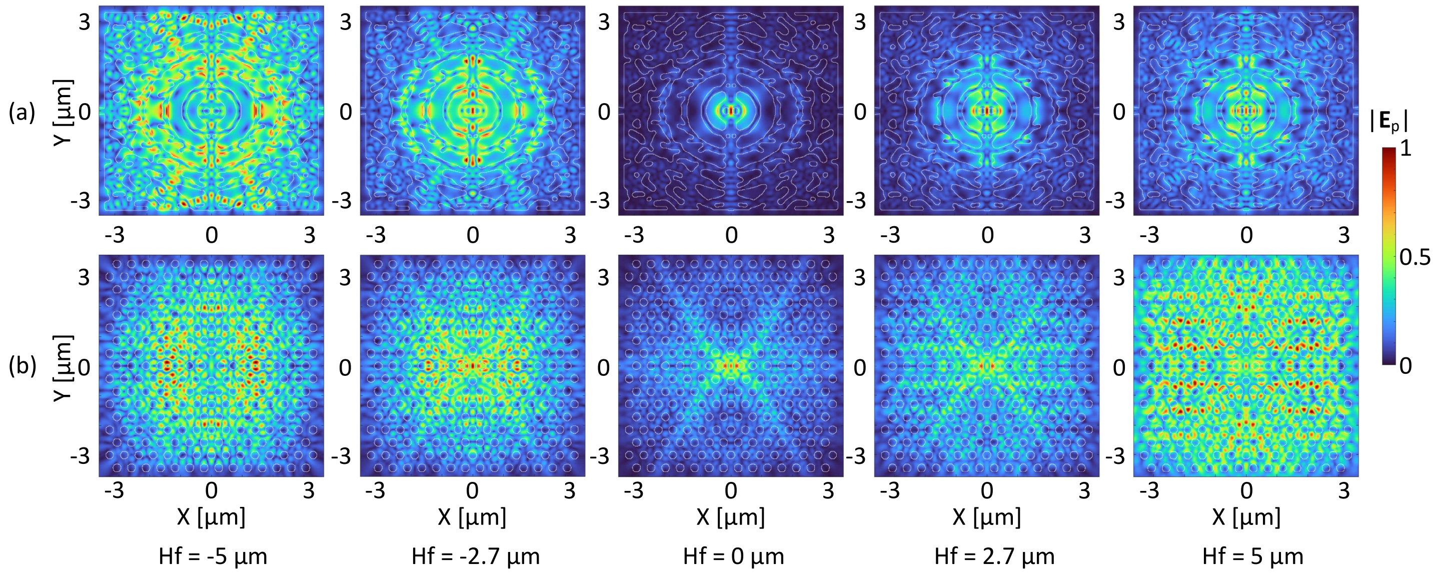

Fig. S9. **Excitation profile of the pump beam at 980 nm.** Same as Fig. S8 but with 980 nm pump light. Excitation patterns for the (a) EDC and (b) PhC nanocavity.

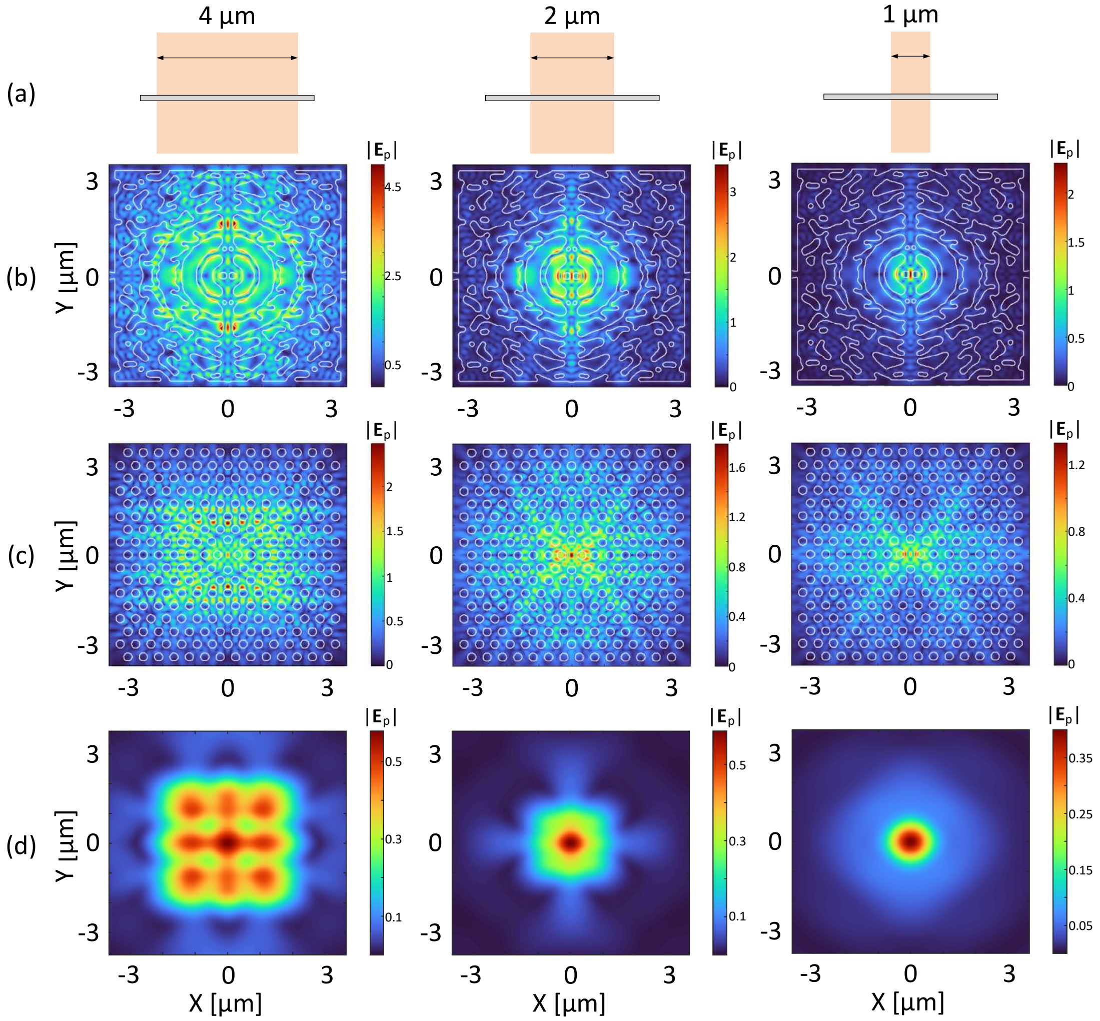

Fig. S10. Excitation profile of a square pump beam at 980 nm. (a) Similar to Fig. S9 but with plane-wave excitation (orange shading) from a square window of varying side lengths. The pump light before striking the device uniformly maintains an intensity of $|\mathbf{E}_p|=1$ across the window. (b) Simulated pump excitation patterns at the central plane of the EDC membrane ($|\mathbf{E}_p|$ not normalized). (c) Same as (b) but for the PhC membrane. (d) Same as (b) and (c) but for a non-structured membrane. From left to right, the side lengths of the pump square window are 4 µm, 2 µm, and 1 µm.

Here, we define the vertical distance between the focal plane of the pump beam and the device's central plane as |Hf|, where positive (negative) Hf means the pump focus is above (below) the membrane, causing a diverging (converging) beam cross the membrane. Fig. S9 shows that at Hf=0, the excited pattern at 980 nm closely resembles the lasing mode, with one antinode at the centre for the EDC and two antinodes near the central holes for the PhC H0 nanocavity.

Fig. S8 shows that the EDC laser exhibits a more dispersed excitation pattern at 1310 nm than the PhC laser, meaning a less localized carrier generation profile. However, the EDC still achieves a lower threshold. Fig. S10 shows the pump pattern from plane-wave injection at varying spot sizes. As the pump spot size decreases, fewer cavity modes are excited, rendering the pattern less homogeneous and increasing the selective excitation of a mode pattern and, subsequently, a carrier distribution that aligns closer with the lasing mode.

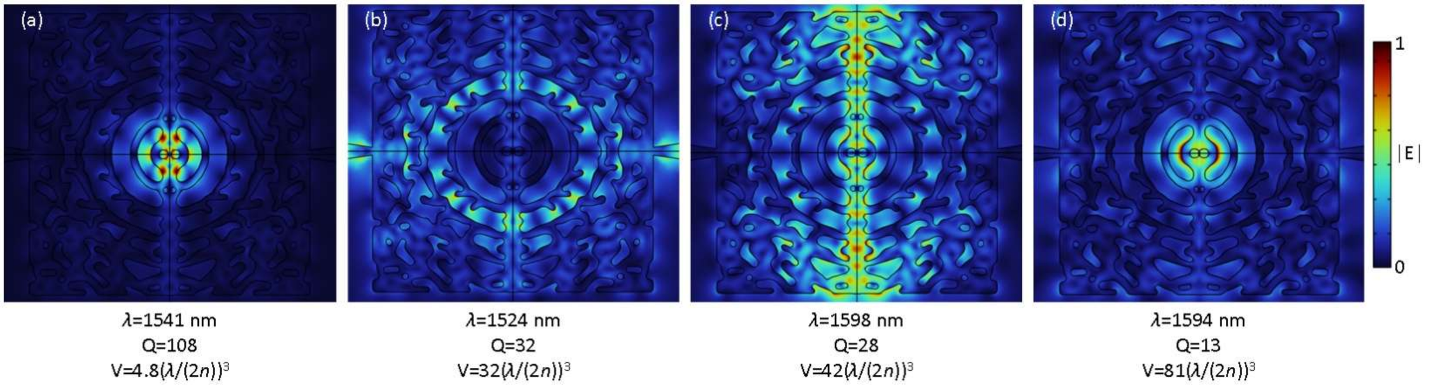

Fig. S11. Characteristic eigenmodes of the EDC cavity beyond the lasing mode. Four modes other than the lasing mode located near the lasing wavelength of the EDC cavity.

To provide a more intuitive understanding of the connection between the excitation pattern and the eigenmodes of the EDC cavity, Fig. S11 presents the additional modes near the lasing wavelength. In addition to the lasing mode at 1530 nm, i.e., the fundamental mode (Fig. 1b in the main text), which has the smallest mode volume and a polarization aligned along the y-axis in the central nanobridge region, the EDC cavity also supports a second low-Q mode at 1541 nm, with Q = 108 and a mode volume of $4.8(\lambda/(2n))^3$ (Fig. S11(a)). The remaining modes exhibit very low Q-factors. We also note that unavoidable, albeit small, reflections at the PML boundaries can introduce additional modes, which are numerical artifacts and are not shown here. As illustrated in Figs. S11(b)-(c), these mode profiles share similarities with the excitation patterns in Fig. 4(b) of the main text, confirming that the excitation pattern is a superposition of eigenmodes. Since no cavity modes exist at our pump laser wavelengths of 980 nm and 1310 nm, the excitation patterns at these wavelengths resemble combinations of the modes around the lasing wavelength.

S5.2. Additional measurement data for EDC and PhC lasers

Figure S12 includes the 980 nm CW pumping measurements and additional measurements of the PhC laser. Fig. S12(b) shows that the PhC laser's threshold curve remains largely unchanged between pulsed and CW pumping (the output power "flattening" at low input power under pulsed pumping is due to noise floor saturation on our spectrum analyzer, resulting from a lower signal-to-noise ratio compared to CW pumping). In contrast, the EDC laser displays a more pronounced S-curve under pulsed pumping (Fig. S12(a)), similar to Fig. 3a, indicating stronger thermal effects due to enhanced carrier and field localization. This pronounced S-curve better aligns with simulation results. The larger thermal effect is further supported by the larger lasing wavelength redshift in the EDC laser (Fig. 2c) compared to the PhC laser (Fig. S12(d)). While surface passivation aids in mitigating the thermal effects, local heating cannot be completely eliminated. Further improvement could be made by encapsulating the device in materials with higher thermal conductivity (64). Although the thermally induced redshift is more pronounced in the EDC laser, it does not degrade its long-term performance, at least within the pump power range used in our experimental setup. We observed that the laser output power remained stable even after several months of measurements.

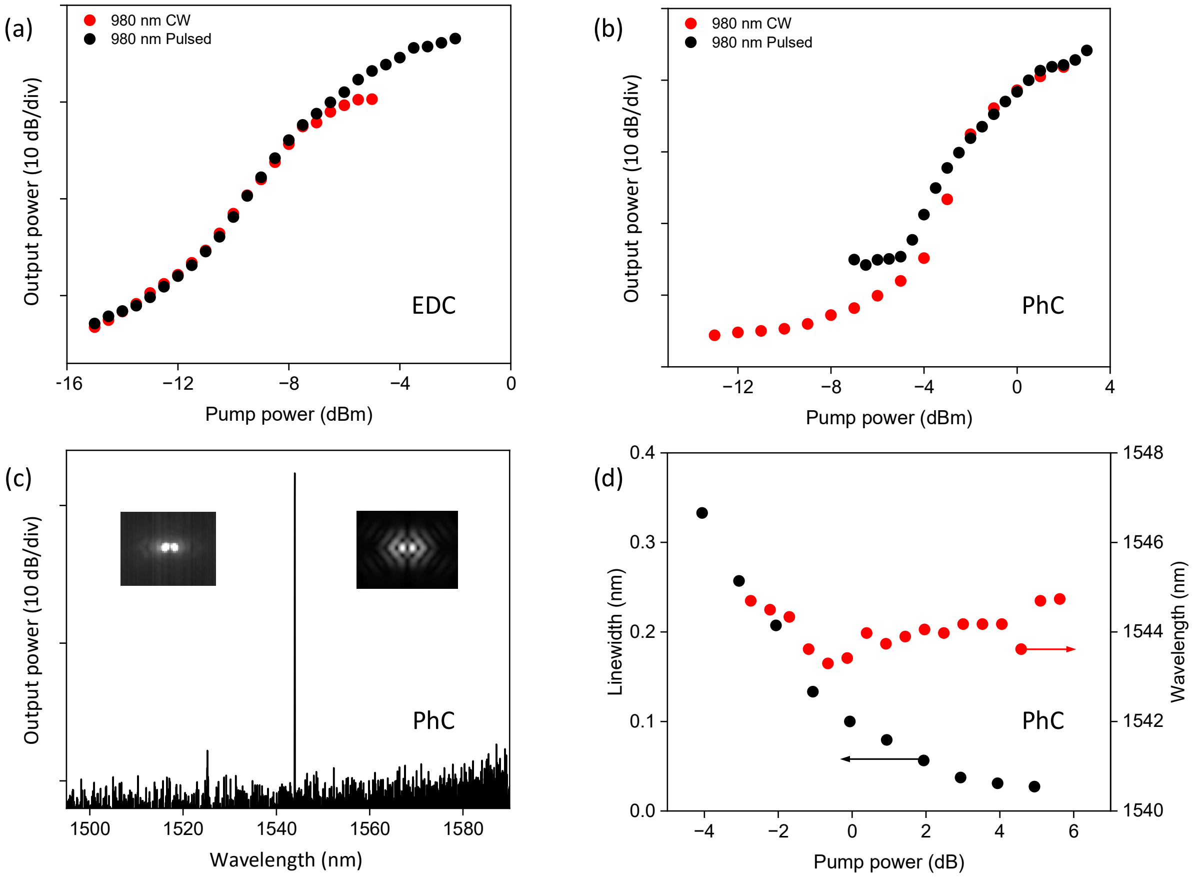

Fig. S12. Additional measurements of the nanolaser threshold characteristics. (a, b) Input-output curves of the EDC (a) and PhC (b) laser under 980 nm CW (red) and pulsed (black) pumping. (c) Lasing spectrum of the PhC H0 laser above threshold. Inset: measured (left) and simulated (right) emission patterns. (d) Measured linewidth (black) and lasing wavelength (red) versus pump power for the PhC H0 laser. The pump power is normalized with zero value at threshold. The linewidth is limited by the resolution (0.02 nm) of our optical spectrum analyzer at high pump powers. In (c) and (d), the pump is the 1310 nm CW light source, as Fig. 2. All measurements are performed at room-temperature.

The highest collected power from the EDC (PhC) laser is around -40 dBm (-48 dBm), with their collection efficiency ratio closely matching simulations. This difference is mainly due to the EDC laser's lower vertical (intrinsic) *Q*-factor and more centralized emission. The EDC laser features a single antinode at the centre (insets in Fig. 2a), in contrast to the dual antinodes of the PhC H0 laser (insets in Fig. S12(c)).

Figures S13 and S14 illustrate the threshold characteristics as the focal height (Hf) varies. It is important to note that both the pumping and collection efficiency decrease with larger |Hf| (e.g., |Hf| = 5 μm), causing a drop in signal-to-noise ratio and reducing the power range with fewer reliable data points. To ensure a more transparent comparison between the lasers, the input and output powers have been normalized, keeping the QW absorbed power (or electrical energy in the QW area) the same for both types, as determined by

$$\int_{\mathrm{EDC},\varepsilon>\varepsilon_0} \varepsilon(x,y)\left|\mathbf{E}_\mathrm{p}(x,y)\right|^2 dxdy = \int_{\mathrm{PhC},\varepsilon>\varepsilon_0} \varepsilon(x,y)\left|\mathbf{E}_\mathrm{p}(x,y)\right|^2 dxdy\,. \tag{S.31}$$

Here, the labels EDC and PhC beneath the integral sign specify that the integration is being performed over the central plane of the respective structures. Noting that by using Eq. (S.31) together with the full-wave simulations to obtain the exact excitation patterns ($\mathbf{E}_\mathrm{p}(x,y)$) at each pump wavelength under the same pumping intensity, the influence of the eigenmode Q-factors on the pumping efficiency is automatically accounted for.

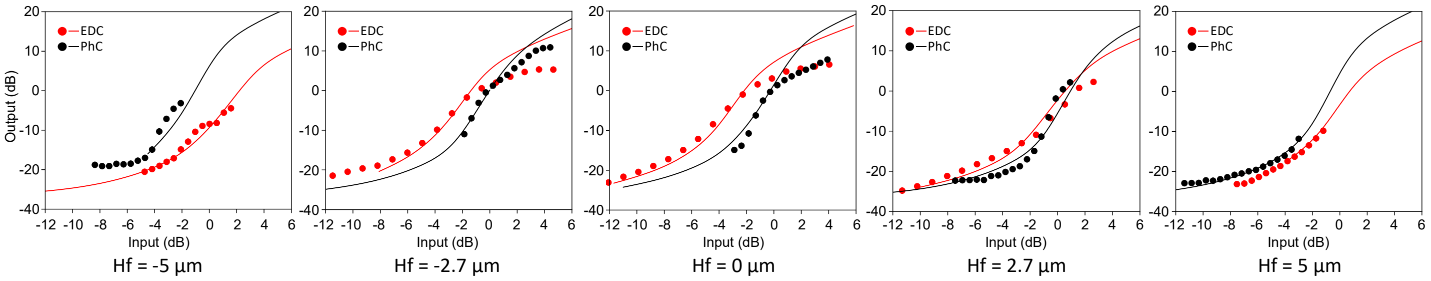

Fig. S13. Input–output curves of the nanolasers under 1310 nm pumping. Threshold characteristics of the EDC (red) and PhC (black) nanolasers for focal heights (Hf) ranging from -5 μm to 5 μm, from left to right. The CW pump at 1310 nm is used. Dots represent experimental data and solid curves show numerical simulations of the 2D laser model. The input power is normalized according to Eq. (S.31), ensuring that the absorbed power is identical for both laser types. Subsequently, both input and output powers are shifted on the log-log scale for clearer comparison across different cases of Hf, while the relative input power between the EDC and PhC lasers is maintained.

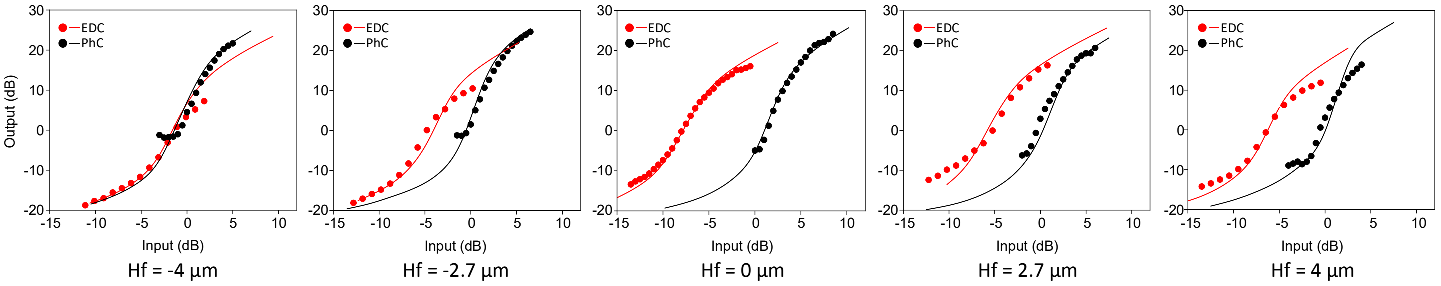

Fig. S14. Input–output curves of the nanolasers under 980 nm pumping. Same as Fig. S13 but with the pulsed pump at 980 nm.

The laser threshold power is determined as the peak of the first derivative of output power with respect to input, calculated on a log-log scale from theoretical fits. This method has proven effective across a wide range of cases (32), (35). Twenty nominally identical devices for each laser type are measured across the wafer. Fig. S15 displays the measurements by varying the focal plane of the pump light, averaged across these 20 devices. Following Eq. (S.31), the threshold values are normalized, with the PhC H0 laser's lowest point set to zero. As seen, both lasers reach a threshold minimum at the focal plane and increase as they move away. Although the threshold reduction for the EDC laser appears smaller at 980 nm before pump power normalization (comparing Figs. 3a and 3b), the reduction is actually larger after the normalization (Fig. 4c).

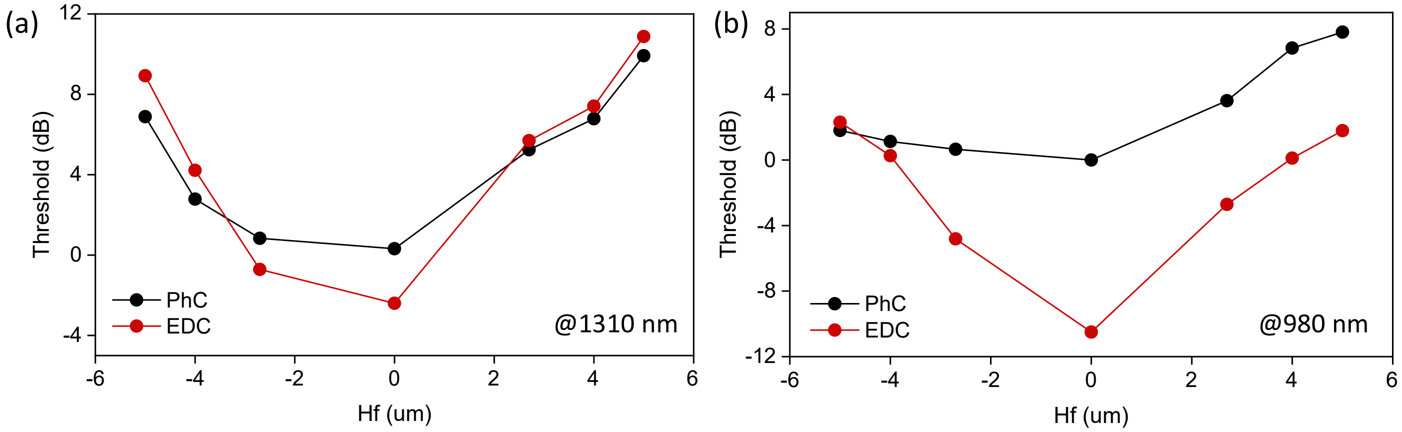

Fig. S15. Variation of the threshold difference as a function of pump focal height. Measured variation in the thresholds of the EDC (red) and the PhC (black) lasers (averaged over 20 devices) for different focal plane positions of the pump light. (a) and (b) correspond to pumping at 1310 nm and 980 nm, respectively. The focal height, Hf, ranges from -5 μm to 5 μm.

We also investigated the sensitivity to lateral shifts of the pump spot and found it to be similar for both the EDC and PhC lasers, consistent with the Gaussian pump spot being much larger than the lateral extent of the optical mode in either case.

S5.3. EDC laser with a narrower dielectric bridge

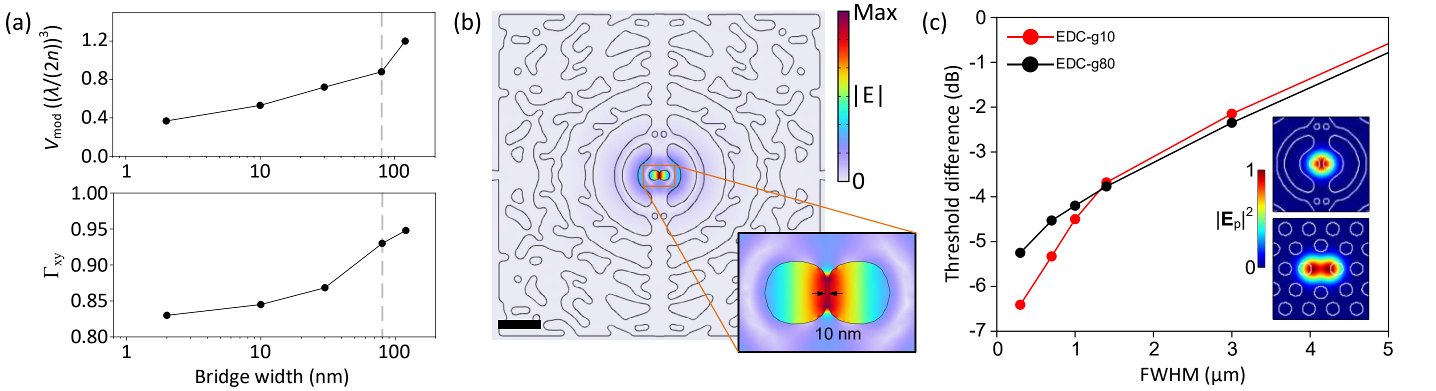

Fig. S16. Effects of dielectric bridge width. (a) Calculated mode volume V_{mod} (upper) and in-plane optical confinement factor Γ_{xy} (lower) versus the nanobridge width in our EDC cavity. The dashed lines mark the structure used. (b) Lasing mode (|**E**|) in the modified EDC cavity (EDC-g10) analogous to the structure (EDC-g80) in Fig. 1, but with a reduced central bridge width of 10 nm. Scale bar: 1 μm. The EDC-g10 is assumed to retain the same resonant wavelength and *Q*-factor as EDC-g80. Inset: zoom-in of the central mode field. (c) Simulated variation in the threshold power difference of the EDC-g10 (red) and EDC-g80 (black) relative to the PhC laser for varying FWHM of the Gaussian excitation pattern ($|\mathbf{E}_p|^2$). The pump power has been normalized according to Eq. (S.31). Insets show the Gaussian excitation patterns for the EDC (upper) and PhC (lower) lasers. The excitation pattern for the EDC lasers is a single Gaussian centred on the EDC mode's "hotspot", while it is a dual-Gaussian peaking at the two antinodes of the PhC H0 nanocavity's fundamental mode.

An advantage of the EDC cavity is its capability to further reduce V_{mod} well below the diffraction limit by simply reducing the feature size of the central dielectric nanostructure (18), (19), as exemplified in Fig. S16(a). However, in our case, reducing the bridge width below 80 nm results in only a gradual decrease in V_{mod} but a sudden drop in the in-plane optical confinement factor Γ_{xy} due to the mode field expansion into the air. Even lower V_{mod} is attainable with tipped or bowtie geometries (18), (19) instead of the bridge form. However, according to our simulations, these geometries are found to accelerate carrier diffusion, increasing V_{car} (and V_{I}) and result in a higher laser threshold.

The EDC laser consistently shows a lower threshold than the PhC laser, especially as the excitation pattern size diminishes (Fig. S16(c)). The EDC-g10 exhibits a higher threshold than EDC-g80 under large pump sizes, likely due to a smaller Γ_{xy}. The advantages of EDC-g10 over EDC-g80 become evident primarily under ultrasmall localized pumping, underscoring the more important roles of V_{car} and V_{I} over V_{mod} to boost light-matter interactions in structures with minimal V_{mod}. Future improvements in EDC laser may include local current injection or other advanced pumping schemes (46), high-density quantum dots with minimized inhomogeneous broadening (65), or buried heterogeneous structures (35) with engineered dimensions. We also performed simulations on structures similar to Refs. (20), (21) where the field is maximized in non-active regions (not shown here), which exhibit higher thresholds than the PhC laser, consistent with findings in Ref. (26). Further analysis will be detailed in future publications.

REFERENCES

1. D. A. B. Miller, Attojoule optoelectronics for low-energy information processing and communications. *J. Lightwave Technol.* **35**, 346–396 (2017).

2. J. Carolan, C. Harrold, C. Sparrow, E. Martín-López, N. J. Russell, J. W. Silverstone, P. J. Shadbolt, N. Matsuda, M. Oguma, M. Itoh, G. D. Marshall, M. G. Thompson, J. C. F. Matthews, T. Hashimoto, J. L. O'Brien, A. Laing, Universal linear optics. *Science* **349**, 711–716 (2015).

3. C. Sun, M. T. Wade, Y. Lee, J. S. Orcutt, L. Alloatti, M. S. Georgas, A. S. Waterman, J. M. Shainline, R. R. Avizienis, S. Lin, B. R. Moss, R. Kumar, F. Pavanello, A. H. Atabaki, H. M. Cook, A. J. Ou, J. C. Leu, Y.-H. Chen, K. Asanović, R. J. Ram, M. A. Popović, V. M. Stojanović, Single-chip microprocessor that communicates directly using light. *Nature* **528**, 534–538 (2015).

4. W. Bogaerts, D. Pérez, J. Capmany, D. A. B. Miller, J. Poon, D. Englund, F. Morichetti, A. Melloni, Programmable photonic circuits. *Nature* **586**, 207–216 (2020).

5. J. Levy, A. Gondarenko, M. A. Foster, A. C. Turner-Foster, A. L. Gaeta, M. Lipson, CMOS-compatible multiple-wavelength oscillator for on-chip optical interconnects. *Nat. Photonics* **4**, 37–40 (2010).

6. N. C. Thompson, K. Greenewald, K. Lee, G. F. Manso, The computational limits of deep learning. arXiv:2007.05558 [cs.LG] (2020).

7. N. Jones, How to stop data centres from gobbling up the world's electricity. *Nature* **561**, 163–166 (2018).

8. E. M. Purcell, Spontaneous emission probabilities at radio frequencies. *Phys. Rev.* **69**, 681 (1946).

9. E. Ozbay, Plasmonics: Merging photonics and electronics at nanoscale dimensions. *Science* **311**, 189–193 (2006).

10. D. Gramotnev, S. Bozhevolnyi, Plasmonics beyond the diffraction limit. *Nat. Photonics* **4**, 83–91 (2010).

11. A. F. Koenderink, A. Alu, A. Polman, Nanophotonics: Shrinking light-based technology. *Science* **348**, 516–521(2015).

12. J. Khurgin, How to deal with the loss in plasmonics and metamaterials. *Nat. Nanotechnol.* **10**, 2–6 (2015).

13. S. Hu, S. M. Weiss, Design of photonic crystal cavities for extreme light concentration. *ACS Photonics* **3**, 1647–1653 (2016).

14. H. Choi, M. Heuck, D. Englund, Self-similar nanocavity design with ultrasmall mode volume for single-photon nonlinearities. *Phys. Rev. Lett.* **118**, 223605 (2024).

15. S. Hu, M. Khater, R. Salas-Montiel, E. Kretschmer, S. Engelmann, W. M. J. Green, S. M. Weiss, Experimental realization of deep-subwavelength confinement in dielectric optical resonators. *Sci. Adv.* **4**, eaat2355 (2018).

16. F. Wang, R. E. Christiansen, Y. Yu, J. Mørk, O. Sigmund, Maximizing the quality factor to mode volume ratio for ultra-small photonic crystal cavities. *Appl. Phys. Lett.* **113**, 241101 (2018).

17. S. Mignuzzi, S. Vezzoli, S. A. R. Horsley, W. L. Barnes, S. A. Maier, R. Sapienza, Nanoscale design of the local density of optical states. *Nano Lett.* **19**, 1613–1617 (2019).

18. M. Albrechtsen, B. V. Lahijani, R. E. Christiansen, V. T. H. Nguyen, L. N. Casses, S. E. Hansen, N. Stenger, O. Sigmund, H. Jansen, J. Mørk, S. Stobbe, Nanometer-scale photon confinement in topology-optimized dielectric cavities. *Nat. Commun.* **13**, 6281 (2022).

19. M. Xiong, R. E. Christiansen, F. Schröder, Y. Yu, L. N. Casses, E. Semenova, K. Yvind, N. Stenger, O. Sigmund, J. Mørk, Experimental realization of deep sub-wavelength confinement of light in a topology-optimized InP nanocavity. *Opt. Mat. Express* **14**, 397–406 (2024).

20. A. N. Babar, T. A. S. Weis, K. Tsoukalas, S. Kadkhodazadeh, G. Arregui, B. V. Lahijani, S. Stobbe, Self-assembled photonic cavities with atomic-scale confinement. *Nature* **624**, 57–63 (2023).

21. Y. Ouyang, H. Luan, Z. Zhao, W. Mao, R. Ma, Singular dielectric nanolaser with atomic-scale field localization. *Nature* **632**, 287–293 (2024).

22. V. R. Almeida, Q. Xu, C. A. Barrios, Lipson, M. Guiding and confining light in void nanostructure. *Opt. Lett.* **29**, 1209–1211 (2004).

23. J. T. Robinson, C. Manolatou, L. Chen, M. Lipson, Ultrasmall mode volumes in dielectric optical microcavities. *Phys. Rev. Lett.* **95**, 143901 (2005).

24. A. Yang, S. Moore, B. Schmidt, M. Klug, M. Lipson, D. Erickson, Optical manipulation of nanoparticles and biomolecules in sub-wavelength slot waveguides. *Nature* **457**, 71–75 (2009).

25. A. D. Falco, L. O'Faolain, T. F. Krauss, Chemical sensing in slotted photonic crystal heterostructure cavities. *Appl. Phys. Lett.* **9**, 063503 (2009).

26. S. Kita, K. Nozaki, S. Hachuda, H. Watanabe, Y. Saito, S. Otsuka, T. Nakada, Y. Arita, T. Baba, Photonic crystal point-shift nanolasers with and without nanoslots—Design, fabrication, lasing, and sensing characteristics. *IEEE J. Sel. Top. Quantum Electron.* **17**, 1632–1647(2011).

27. P. Seidler, K. Lister, U. Drechsler, J. Hofrichter, T. Stöferle, Slotted photonic crystal nanobeam cavity with an ultrahigh quality factor-to-mode volume ratio. *Opt. Express* **21**, 32468–32483 (2013).

28. H. Wu, L. Yang, P. Xu, J. Gong, X. Guo, P. Wang, L. Tong, Photonic nanolaser with extreme optical field confinement. *Phys. Rev. Lett.* **129**, 013902 (2022).

29. X. Ding, Y. He, Z. Duan, N. Gregersen, M. Chen, S. Unsleber, S. Maier, C. Schneider, M. Kamp, S. Hofling, C. Lu, J. Pan, On-demand single photons with high extraction efficiency and near-unity indistinguishability from a resonantly driven quantum dot in a micropillar. *Phys. Rev. Lett.* **116**, 020401 (2016).

30. P. Senellart, G. Solomon, A. White, High-performance semiconductor quantum-dot single-photon sources. *Nat. Nanotechnol.* **12**, 1026–1039 (2017).

31. L. Coldren, S. Corzine, *Diode Lasers and Photonic Integrated Circuits* (Wiley, ed. 2, 2012).

32. M. Saldutti, Y. Yu, J. Mørk, The onset of lasing in semiconductor nanolasers. *Laser Photonics Rev.* **18**, 2300840 (2024).

33. S. Matsuo, A. Shinya, T. Kakitsuka, K. Nozaki, T. Segawa, T. Sato, Y. Kawaguchi, M. Notomi, High-speed ultracompact buried heterostructure photonic-crystal laser with 13 fJ of energy consumed per bit transmitted. *Nat. Photonics* **4**, 648–654 (2010).

34. Y. Yu, A. Sakanas, A. R. Zali, E. Semenova, K. Yvind, J. Mørk, Ultra-coherent Fano laser based on a bound state in the continuum. *Nat. Photonics* **15**, 758–764 (2021).

35. E. Dimopoulos, M. Xiong, A. Sakanas, A. Marchevsky, G. Dong, Y. Yu, E. Semenova, J. Mørk, K. Yvind, Experimental demonstration of nanolaser with sub-μA threshold current. *Optica* **10**, 973–976 (2023).

36. G. Kountouris, J. Mørk, E. V. Denning, P. T. Kristensen, Modal properties of dielectric bowtie cavities with deep sub-wavelength confinement. *Opt. Express* **30**, 40367–40378 (2022).

37. E. Kapon, S. Simhony, R. Bhat, D. M. Hwang, Single quantum wire semiconductor lasers. *Appl. Phys. Lett.* **55**, 2715–2717 (1989).

38. G. Crosnier, A. Bazin, V. Ardizzone, P. Monnier, R. Raj, F. Raineri, Subduing surface recombination for continuous-wave operation of photonic crystal nanolasers integrated on silicon waveguides. *Opt. Express* **23**, 27953–27959 (2015).

39. N. M. Andrade, S. Hooten, Y. Kim, J. Kim, E. Yablonovitch, M. C. Wu, Sub-50 cm/s surface recombination velocity in InGaAsP/InP ridges. *Appl. Phys. Lett.* **119**, 191102 (2021).

40. A. Higuera-Rodriguez, B. Romeira, S. Birindelli, L. E. Black, E. Smalbrugge, P. J. van Veldhoven, W. M. M. Kessels, M. K. Smit, A. Fiore, Ultralow surface recombination velocity in passivated InGaAs/InP nanopillars. *Nano Lett.* **17**, 2627–2633 (2017).

41. Z. Zhang, M. Qiu, Small-volume waveguide-section high *Q* microcavities in 2D photonic crystal slabs. *Opt. Express* **12**, 3988–3995 (2004).

42. Y. Yu, W. Xue, E. Semenova, K. Yvind, J. Mørk, Demonstration of a self-pulsing photonic crystal Fano laser. *Nat. Photonics* **11**, 81–84 (2017).

43. Y. Yu, E. Palushani, M. Heuck, N. Kuznetsova, P. T. Kristensen, S. Ek, D. Vukovic, C. Peucheret, L. K. Oxenløwe, S. Combrié, A. de Rossi, K. Yvind, J. Mørk, Switching characteristics of an InP photonic crystal nanocavity: Experiment and theory. *Opt. Express* **21**, 31047–31061 (2013).

44. M. Saldutti, Y. Yu, G. Kountouris, P. T. Kristensen, J. Mørk, Carrier diffusion in semiconductor nanoscale resonators. *Phys. Rev. B* **109**, 245301 (2024).

45. C. Roques-Carmes, N. Rivera, A. Ghorashi, S. E. Kooi, Y. Yang, Z. Lin, J. Beroz, A. Massuda, J. Sloan, N. Romeo, Y. Yu, J. D. Joannopoulos, I. Kaminer, S. G. Johnson, M. Soljacic, A framework for scintillation in nanophotonics. *Science* **375**, eabm9293 (2022).

46. K. Yoshida, J. Gong, A. L. Kanibolotsky, P. J. Skabara, G. A. Turnbull, I. D. W. Samuel, Electrically driven organic laser using integrated OLED pumping. *Nature* **621**, 746–752 (2023).

47. G. Konstantatos, E. Sargent, Nanostructured materials for photon detection. *Nat. Nanotechnol.* **5**, 391–400 (2010).

48. K. Nozaki, S. Matsuo, T. Fujii, K. Takeda, M. Ono, A. Shakoor, E. Kuramochi, M. Notomi, Photonic-crystal nano-photodetector with ultrasmall capacitance for on-chip light-to-voltage conversion without an amplifier. *Optica* **3**, 483–492 (2016).

49. M. Ayata, Y. Fedoryshyn, W. Heni, B. Baeuerle, A. Josten, M. Zahner, U. Koch, Y. Salamin, C. Hoessbacher, C. Haffner, D. L. Elder, L. R. Dalton, J. Leuthold, High-speed plasmonic modulator in a single metal layer. *Science* **358**, 630–632 (2017).

50. H. Pahlevaninezhad, M. Khorasaninejad, Y.-W. Huang, Z. J. Shi, L. P. Hariri, D. C. Adams, V. Ding, A. Zhu, C. W. Qiu, F. Capasso, M. J. Suter, Nano-optic endoscope for high-resolution optical coherence tomography in vivo. *Nat. Photonics* **12**, 540–547 (2018).

51. K. Frischwasser, K. Cohen, J. Kher-Alden, S. Dolev, S. Tsesses, G. Bartal, Real-time sub-wavelength imaging of surface waves with nonlinear near-field optical microscopy. *Nat. Photonics* **15**, 442–448 (2021).

52. Y. H. Chou, K. B. Hong, C. T. Chang, T. C. Chang, Z. T. Huang, P. J. Cheng, J. H. Yang, M. H. Lin, T. R. Lin, K. P. Chen, S. Gwo, T. C. Lu, Ultracompact pseudowedge plasmonic lasers and laser arrays. *Nano Lett.* **18**, 747–753 (2018).

53. J. Y. Suh, C. H. Kim, W. Zhou, M. D. Huntington, D. T. Co, M. R. Wasielewski, T. W. Odom, Plasmonic bowtie nanolaser arrays. *Nano Lett.* **12**, 5769–5774 (2010).

54. S. H. Kwon, J. H. Kang, C. Seassal, S. K. Kim, P. Regreny, Y. H. Lee, C. M. Lieber, H. G. Park, Subwavelength plasmonic lasing from a semiconductor nanodisk with silver nanopan cavity. *Nano Lett.* **10**, 3679–3683 (2010).

55. S. Jiang, D. Belogolovskii, S. S. Deka, S. H. Pan, Y. Fainman, Experimental demonstration of mode selection in bridge-coupled metallo-dielectric nanolasers. *Opt. Lett.* **46**, 6027–6030 (2021).

56. J. Xu, T. Zhang, Y. Li, J. Zhang Z. Wang, Q. Kan, R. Zhang, C.-Z. Ning, Room-temperture low-threshold plasmonic nanolaser through mode-tailoring at communication wavelengths. *Laser Photonics Rev.* **17**, 2200322 (2023).

57. K. Ding, Z. C. Liu, L. J. Yin, M. T. Hill, M. J. H. Marell, P. J. van Veldhoven, R. Nöetzel, C. Z. Ning, Room-temperature continuous wave lasing in deep-subwavelength metallic cavities under electrical injection. *Phys. Rev. B* **85**, 041301 (2012).

58. E. Fermi, Quantum theory of radiation. *Rev. Mod. Phys.* **4**, 87–132 (1932).

59. K. Nozaki, S. Kita, T. Baba, Room temperature continuous wave operation and controlled spontaneous emission in ultrasmall photonic crystal nanolaser. *Opt. Express* **15**, 7506–7514 (2007).

60. K. Nozaki, T. Baba, Carrier and photon analyses of photonic microlasers by two-dimensional rate equations. *IEEE J Sel Areas Commun* **23**, 1411–1417 (2005).

61. H. Wenzel, M. Kantner, M. Radziunas, U. Bandelow, Semiconductor laser linewidth theory revisited. *Appl. Sci.* **11**, 6004 (2021).

62. W. S. Rabinovich, B. J. Feldman, Spatial hole burning effects in distributed feedback lasers. *IEEE J. Quantum Electron.* **25**, 20–30 (1989).

63. J. Mørk, G. L. Lippi, Rate equation description of quantum noise in nanolasers with few emitters. *Appl. Phys. Lett.* **112**, 141103 (2018).

64. A. Bazin, P. Monnier, X. Lafosse, G. Beaudoin, R. Braive, I. Sagnes, R. Raj, F. Raineri, Thermal management in hybrid InP/silicon photonic crystal nanobeam laser. *Opt. Express* **22**, 10570–10578 (2014).

65. S. Banyoudeh, J. P. Reithmaier, High-density 1.54 μm InAs/InGaAlAs/InP(100) based quantum dots with reduced size inhomogeneity. *J. Cryst. Growth*, **425**, 299–302 (2015).
